# Plasma multi-miRNA models classify Alzheimer’s, Parkinson’s, and Lewy body dementia

**DOI:** 10.3389/fnagi.2026.1788991

**Published:** 2026-08-11

**Authors:** Ursula S. Sandau, Jack T. Wiedrick, Trevor J. McFarland, Dora Yearout, Cyrus P. Zabetian, Shu-Ching Hu, Debby W. Tsuang, Joseph F. Quinn, Julie A. Saugstad

**Affiliations:** 1 Department of Anesthesiology & Perioperative Medicine, Oregon Health & Science University, Portland, OR, United States; 2 Biostatistics & Design Program, Oregon Health & Science University, Portland, OR, United States; 3 Geriatric Research, Education, and Clinical Center, VA Puget Sound Health Care System, Seattle, WA, United States; 4 Department of Neurology, University of Washington, Seattle, WA, United States; 5 Department of Psychiatry and Behavioral Sciences, University of Washington, Seattle, WA, United States; 6 Department of Neurology, Oregon Health & Science University, Portland, OR, United States; 7 Parkinson Center and Movement Disorders Program, Oregon Health & Science University, Portland, OR, United States; 8 Portland VAMC Parkinson's Disease Research, Education, and Clinical Center, Portland, OR, United States

**Keywords:** Alzheimer’s disease, human, Lewy body dementia, microRNA, Parkinson’s disease, plasma, predictive modeling, sex differences

## Abstract

**Introduction:**

Differentiating Alzheimer’s disease (AD) from both Parkinson’s disease (PD) and Lewy body dementia (DLB) is difficult due to their clinical similarities. Plasma biomarkers offer an alternative to neuroimaging and cerebrospinal fluid analysis; however, there are limitations with respect to differential diagnosis of AD, PD, and DLB with current clinical assays.

**Methods:**

Here we used machine learning to assess plasma miRNAs for their specificity in diagnosing AD vs. PD, and DLB. Our multi-center study assayed 57 AD-associated miRNAs in human plasma from 82 cognitively normal controls (NC), 87 AD, 100 PD, and 20 DLB. Predictive models generated by three independent machine learning methods were evaluated by cross-validated ROC curves [cvAUC (bootstrap bias-corrected 95% CI)]. We also used linear discriminant analysis with all 57 miRNAs to identify a model that best separates AD from PD + DLB participants and DLB from AD+PD participants. Further, we used Target prediction and Ingenuity Pathway Analysis to identify highly relevant mRNA targets of the miRNAs.

**Results:**

Individual assessment of the 57 miRNAs identified a subset of 10 miRNAs that were more AD-specific, and 23 miRNAs that were more PD and/or DLB associated. Ridge logistic regression predictive models with the 10 AD-specific miRNAs had good performance for separating AD vs. PD and DLB (cvAUC = 0.77 [0.70, 0.83]) and AD vs. PD (cvAUC = 0.79 [0.71, 0.85]), but not for AD vs. DLB (cvAUC = 0.58 [0.43, 0.79]). By developing a predictive model using data from all 57 miRNAs and elastic-net regression we achieved good separation of AD from PD (cvAUC = 0.80 [0.72, 0.86]) and DLB (cvAUC = 0.77 [0.64, 0.87]) with a subset of six miRNAs (miRs-19a-3p, 22-3p, 92b-3p, 101-3p, 143-3p, 423–5p) identified as the most important to these models. The linear discriminant analysis model achieved very good classification of AD vs. PD + DLB (cvAUC = 0.94 [0.87, 0.97]), PD from AD+DLB (cvAUC = 0.88 [0.80, 0.92]), and DLB from AD+PD (cvAUC = 0.85 [0.78, 0.89]) with miR-26a-5p and 146a-5p being most important for AD vs. PD + DLB, and miR-142-3p and 101-3p being most important for DLB vs. AD+PD. Target prediction and Ingenuity Pathway Analysis with miR-26a-5p, 146a-5p, 142-3p, 101-3p returned highly relevant mRNA targets associated with tauopathy, dementia, and movement disorders.

**Discussion:**

These data demonstrate that predictive modeling using plasma miRNA expression data may improve the differential diagnosis of AD from PD from DLB.

## 1 Introduction

Biomarker research for the diagnosis of Alzheimer’s disease (AD) is challenging due to clinical similarities and co-pathologies that exist in AD, Parkinson’s disease (PD), and Lewy body dementia (DLB). Dopamine transporter scans (Nihashi et al., 2020) and cerebrospinal fluid (CSF) α-synuclein seed amplification assays (Arnold et al., 2022) can be helpful for distinguishing DLB from AD, but both are less useful for distinguishing DLB from PD and are dependent on technology and expertise that is not widely available. Plasma biomarkers of neurodegenerative disease have been touted as non-invasive, widely available alternatives to neuroimaging and CSF analysis (Quinn and Gray, 2024), but have limitations with respect to DLB. For example, the α-synuclein seed amplification assay has greatly limited sensitivity in blood (Christenson et al., 2024), and plasma biomarkers of AD pathology are positive in a portion of people with DLB (Alam et al., 2023), reflecting the frequency of dual amyloid-tau and α-synuclein pathology in many of these patients (Sierra et al., 2016). There is consequently a need for plasma biomarkers that can distinguish AD from PD and DLB. Here, we present a panel of plasma miRNAs which have the potential to serve this purpose.

Mature miRNAs are small, non-coding RNAs that regulate mRNA translation via binding to sequences in the 3’UTR of target mRNAs (Kapplingattu et al., 2025). MiRNAs modulate many aspects of neurophysiology including plasticity and cognitive function (Blount et al., 2022), while aberrant miRNA levels can contribute to the development of amyloid (Aβ), tau, and α-synuclein pathology (Blount et al., 2022; Kapplingattu et al., 2025). For example, miR-101-3p directly regulates amyloid precursor protein (Vilardo et al., 2010), and also contributes to α-synuclein aggregation and toxicity in cultured neurons (Zhang et al., 2021). In humans with autopsy-confirmed AD, DLB, and AD+DLB, miRNA expression is both pathology and brain region dependent (Wang et al., 2011; Hebert et al., 2013; Nelson et al., 2018; Luo et al., 2025) with ~85% of miRNA changes in the dorsolateral prefrontal cortex specific to either AD or DLB pathology (Luo et al., 2025), and miRNA levels correlate with burden of Aβ plaques, neurofibrillary tangles, or neuritic plaques in the temporal cortex (Wang et al., 2011). Importantly, AD-dependent changes in brain miRNAs are detectable in antemortem CSF and plasma (Bekris et al., 2013). Further, CSF and serum miRNAs correlate with Braak stage, neurofibrillary tangle score, Aβ plaque density, and Lewy body stage (Burgos et al., 2014). Consequently, there is great interest in miRNAs as biomarkers of AD, PD, and DLB (Blount et al., 2022).

Previous studies have compared blood miRNAs in AD to DLB, and only one has compared AD to DLB and PD (Sorensen et al., 2016; Gamez-Valero et al., 2019; Shigemizu et al., 2019a; Shigemizu et al., 2019b; Asanomi et al., 2021; Jia et al., 2021; Li et al., 2022). Four studies used machine learning in Asian cohorts to develop multi-miRNA prediction models that classify AD or DLB vs. normal controls (NC) (AUC > 0.8) (Shigemizu et al., 2019a; Shigemizu et al., 2019b; Asanomi et al., 2021; Jia et al., 2021). One assessed disease discrimination but did not achieve good performance (mean accuracy = 0.38; Asanomi et al., 2021). Another showed that a combination of seven plasma miRNAs that was predictive of CSF p-tau and Aβ_42_ measurements in AD vs. NC had good performance for AD vs. five pooled groups: NC, DLB, PD with dementia (PDD), vascular dementia (VaD), and frontal temporal dementia (FTD; AUC = 0.87; Jia et al., 2021). Here we used our established miRNA analysis pipeline to investigate the potential of predictive models to classify AD vs. PD vs. DLB. Previously, we used machine learning to generate a 14-miRNA model for AD vs. NC that together with CSF Aβ_42_:total tau improved the performance of clinical CSF AD markers (AUC = 0.903; Lusardi et al., 2017; Wiedrick et al., 2019). Recently, we examined the classification performance of 57 miRNAs previously prioritized in our studies (Lusardi et al., 2017; Wiedrick et al., 2019; Sandau et al., 2020; Sandau et al., 2024) and in studies by other groups (Nagaraj et al., 2017; Qian et al., 2019; Awuson-David et al., 2023) as candidate AD biomarkers in donor- and date-matched CSF and plasma from 160 participants (Sandau et al., 2025). We showed that models based on either CSF or plasma miRNAs had similar performance in a discovery cohort, but only the plasma models maintained performance in a validation cohort (Sandau et al., 2025). Further, the plasma miRNA models plus clinical predictors (e.g., *APOE4*) and CSF Aβ_42_:total tau data achieved excellent classification performance (AUC = 0.958) (Sandau et al., 2025). We now present results from a multi-center study with 289 participants that show plasma multi-miRNA models generated by two independent machine-learning methods have robust classification for AD vs. PD and/or DLB. Furthermore, pathway analyses with four miRNAs prioritized in a linear discriminant (LD) analysis returned highly relevant mRNA targets associated with tauopathy, dementia, movement disorders, and sleep disorders (although the association of these miRNAs with sleep disorders is limited to target prediction, as this dataset did not include standardized sleep questionnaires or polysomnography). Together, these findings demonstrate the potential of plasma miRNAs to bring new information for differential diagnosis of AD, DLB, and PD that is relevant to clinical features.

## 2 Methods

### 2.1 Participant samples

**Figure 1:**
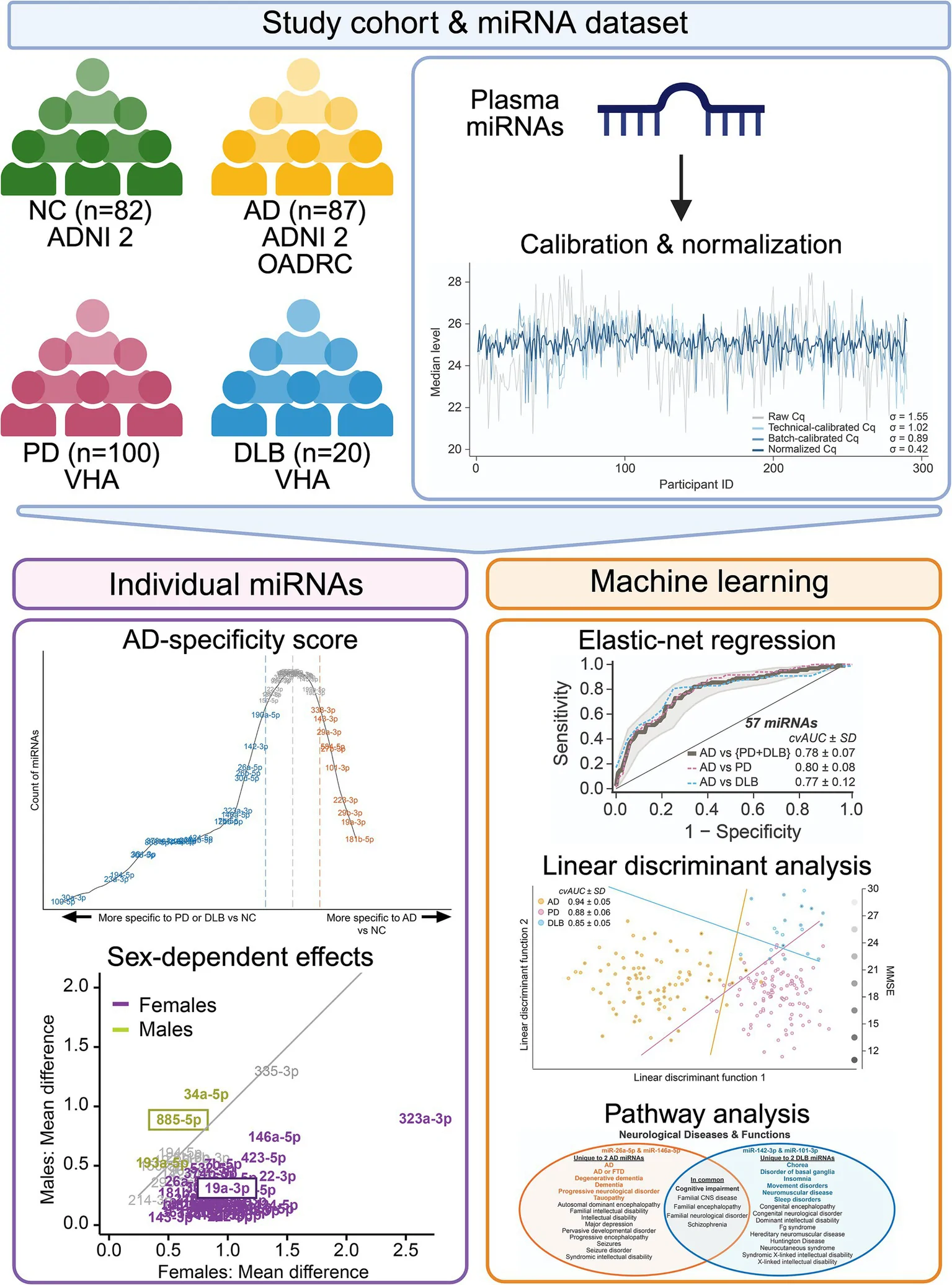
Study design schematic. The multi-center study included human plasma from the Alzheimer’s Disease Neuroimaging Initiative 2 (ADNI 2) cohort, the Oregon Alzheimer’s Disease Research Center (OADRC), and Veterans Health Administration (VHA) Puget Sound Health Care System. Study cohort groups included cognitively normal controls (NC, *n* = 82), AD (*n* = 87), PD (*n* = 100), and DLB (*n* = 20). RT-qPCR was used to quantify plasma expression levels of 64 miRNAs that included 57 AD-associated miRNAs, 1 spike-in miRNA for exogenous calibration, 4 endogenous control miRNAs for normalization, and 2 negative control miRNAs. The 57 miRNAs were assessed in individuals to determine an AD-specificity score and to examine the sex-dependent effects on miRNA changes in AD. Machine learning via ENR and LD analysis was employed to develop predictive models for AD vs. PD vs. DLB using data for all 57 miRNAs. Target prediction and pathway analysis using IPA (Ingenuity Pathway Analysis) software was performed on the top-2 miRNAs most important to AD vs. PD + DLB classification compared to the top-2 miRNAs most important to DLB vs. AD+PD classification.

A schematic of our study design is provided in Figure 1. Participant samples and data were obtained from the Alzheimer’s Disease Neuroimaging Initiative 2 (ADNI 2), the Oregon Alzheimer’s Disease Research Center (OADRC), and the Veterans Health Administration (VHA) Puget Sound Health Care System. ADNI data used in the preparation of this article were obtained from the ADNI database.^1^ The ADNI project was launched in 2003 as a public-private partnership (Aisen et al., 2024). The primary goal of ADNI has been to test whether serial magnetic resonance imaging (MRI), positron emission tomography (PET), other biological markers, and clinical and neuropsychological assessment can be combined to measure the progression of mild cognitive impairment (MCI) and early Alzheimer’s disease (AD) (Aisen et al., 2024). For this study, we obtained plasma samples from 83 healthy cognitive controls (NC, ADNI 2), 87 AD (77 ADNI 2 + 10 OADRC), 100 PD (VHA), and 20 DLB (VHA), *n* = 290 total. The PD group included 49 with cognitive impairment and no dementia (PD-MCI), 12 with dementia (PDD), and 39 with no cognitive impairment (PD-NCI). Of the total 290 samples, one NC male plasma sample had considerable hemolysis (very pink plasma) and was excluded from analysis. Thus, the study included miRNA data from *n* = 289 plasma samples.

Samples were collected in a uniform fashion in EDTA tubes, with plasma separated and frozen at −80 °C until analysis. The DLB sample size was limited to n = 20 because of sample availability. All studies using human samples were approved by the OHSU Institutional Review Board Committee, IRB #00009707. We certify that the study was performed in accordance with the ethical standards as laid down in the 1964 Declaration of Helsinki and its later amendments and all participants provided informed consent.

### 2.2 Participant cohort and cognitive data

NC participants were free of memory complaints, 96% scored between 24 and 30 on the Mini-Mental State Examination (MMSE), and all displayed normal memory function as assessed by the Wechsler Memory Scale. Participants with AD met the original National Institute of Neurological and Communicative Disorders and Stroke/Alzheimer’s Disease and Related Disorders Association inclusion criteria for probable AD revised criteria (Jack et al., 2011). Participants with PD met the International Parkinson and Movement Disorder Society criteria of bradykinesia plus rest tremor or rigidity, in the absence of any exclusionary criteria (Postuma et al., 2018). Participants with DLB met the criteria for a clinical diagnosis of probable or possible DLB as revised by the DLB Consortium in 2017 (McKeith et al., 2017).

Study variables on all participants included: age at time of plasma donation, self-reported sex (categorized as female or male only; accepted as a proxy for chromosomal sex designation), MMSE scores for NC and AD participants, and Montreal Cognitive Assessment (MoCA) scores for PD and DLB participants (Table 1). To compare cognitive status between AD, PD, DLB, and NC, the raw MoCA scores for PD and DLB participants were converted to a weighted equivalent MMSE score (Fasnacht et al., 2023). Age at time of plasma donation and sex were adjusted in all statistical analyses as it was not possible to balance the diagnosis groups on these two factors due to sample availability constraints.

**Table 1:** Participant demographics for each diagnostic group include the number of participants, mean age of participants at time of blood draw, number of females and males, and mean MMSE scores. MMSE scores are listed for NC and AD, while PD and DLB were converted from MoCA scores (Fasnacht et al., 2023) and are thus reported as MMSE-equivalent scores. Significant group differences were identified for age, sex, and MMSE scores. Standardized differences including standardized mean differences or standardized count differences were used for comparisons between each disease diagnostic group for age at time of blood draw, sex, and MMSE scores. Large standardized differences (bold font, absolute values > ~0.3) were considered informative for discriminating group differences (Cohen, 1988; Parast et al., 2020).

*n* (% of cohort) mean ± SD [range]	NC	AD	PD	DLB	*p*
Number of participants	82 (28.4%)	87 (30.1%)	100 (34.6%)	20 (6.9%)	
Age at time of blood draw	73.80 ± 7.08 [56,89]	74.77 ± 8.53 [54,91]	67.61 ± 8.27 [48,88]	71.55 ± 6.92 [55,87]	<0.001
Sex					0.011
Female	39 (47.6%)	37 (42.5%)	36 (36.0%)	2 (10.0%)	
Male	43 (52.4%)	50 (57.5%)	64 (64.0%)	18 (90.0%)	
MMSE for NC/AD, MMSE-equivalent for PD/DLB	28.49 ± 1.87 [21,30]	22.48 ± 2.96 [10,26]	28.08 ± 1.88 [20,30]	21.70 ± 5.19 [11,29]	<0.001

Standardized differences						
	AD vs. NC	PD vs. NC	DLB vs. NC	AD vs. PD	AD vs. DLB	PD vs. DLB
Age	0.12	**−0.81**	**−0.32**	**0.85**	**0.41**	**−0.52**
Sex	0.10	0.23	**0.90**	−0.13	**−0.79**	**−0.64**
MMSE	**−2.43**	−0.22	**−1.74**	**−2.26**	0.19	**1.63**

Large standardized differences (bold font, absolute values > ~0.3) were considered informative for discriminating group differences.

### 2.3 Selection of miRNAs for custom array panel

The 64 miRNAs included in the custom array were selected based on our published AD miRNA biomarker studies in plasma and CSF data and a literature search (Lusardi et al., 2017; Nagaraj et al., 2017; Qian et al., 2019; Wiedrick et al., 2019; Sandau et al., 2020; Awuson-David et al., 2023; Sandau et al., 2024; Sandau et al., 2025) (Supplementary Table 1A). The selected miRNAs fell into four categories: (1) 57 miRNAs that had prior evidence of association (increased or decreased, in most cases repeatedly and consistently across prior experiments) with dementia severity and predictive power for discriminating AD from NC individuals either singly or (more often) in combination with other miRNAs; (2) four miRNAs that are reliably measurable in plasma and confirmed by our experience to be unassociated with AD, for use as endogenous normalizer controls because of their known stable expression across different samples;^2^ (3) two miRNAs that are known to be lacking expression in plasma and for use here as negative controls; and (4) one exogenous spike-in positive control (cel-miR-39-3p) for Cq calibration. Details regarding the assays (miRBase and Thermo Fisher Scientific (TFS) probes) are presented in Supplementary Table 1B. In order to improve reliability of the RT-qPCR, each miRNA was run in triplicate on a custom 384-well card that included simultaneous measurement of samples from two participants (64 selected miRNAs x 3 replicate wells x 2 participant samples = 384 wells). Detailed information regarding each miRNA probe in the array and their performance in the assay is provided in Supplementary Table 1C.

### 2.4 RNA isolation and miRNA RT-qPCR profiling

The 289 plasma samples were randomized prior to sample processing, and the operator was blinded to their diagnosis. Total RNA was isolated from 100 μL of plasma using the MagMax Mirvana Total RNA Isolation Kit (TFS) using a KingFisher Apex System (TFS) automated purification instrument following the manufacturer’s protocol. For the RNA isolation, the 289 plasma samples were processed in a total of 4 batches (3 plates x 80 samples = 240, 1 plate x 49 samples). On the first day, plasma samples were mixed with 5 μL of proteinase K and 45 μL of digestion buffer and incubated for 30 min at 65 °C; 100 μL of lysis buffer containing 1% *β*-mercaptoethanol was added to each well, mixed and stored at −20 °C overnight. The following day, the plate was thawed to room temperature and an exogenous cel-miR-39-3p spike-in control (TFS) was added at a final concentration of 3 pM, followed by the addition of 20 μL of magnetic isolation beads. Samples were mixed for 5 min using a plate shaker followed by the addition of 270 μL of isopropanol and brief mix. The sample plate as well as 4 wash plates (each containing 150 μL of wash buffer), a DNase plate containing 45 μL of Turbo DNase buffer and 2 μL of DNase, and an elution plate containing 50 μL of nuclease free water were all processed using the KingFisher Apex instrument. Following the DNase step, 50 μL re-binding buffer and 100 μL of isopropanol was added to each well and automation was resumed for the washing and elution steps. All isolations were completed within two consecutive days. Following the elution step, the miRNA concentration of a subset of samples from each batch (minimum of 30% of samples per batch across each plate) was measured using the Qubit miRNA Assay kit (TFS) and read using a Qubit 4.0 fluorometer (TFS) to ensure the isolation was consistent across the batch. Prior to the eluted RNA being frozen at −80 °C until RT-qPCR, each plate of 80 RNA samples was divided into 2 plates of 40 RNA samples, as 40 is the maximum number of miRNA RT-qPCR assays that can be processed in a day, and the plate with 49 RNA samples was divided into 1 plate of 40 samples and 1 plate of 9 samples. Thus, the RT-qPCR was performed with the 289 eluted RNA samples in a total of 8 batches (7 × 40 RNA = 280, 1 × 9 RNA), with one batch being completed per day in unbroken succession to mitigate time drift in measurements.

The RT-qPCR was performed in the order of RNA isolation. MiRNA was converted to cDNA as previously described (Sandau et al., 2024) using the TaqMan Advanced miRNA cDNA Synthesis kit (TFS). Briefly, 3 μL of total RNA was 3′ poly-adenylated, followed by a 5′ adaptor ligation step and reverse transcription. The resulting cDNA (5 μL) was added to a 14-cycle universal miR-amplification step. The miR-amplification reaction was diluted 1:10 with nuclease free water, 220 μL of the diluted cDNA was mixed with 220 μL of nuclease free water and 440 μL of TaqMan Fast Advanced master mix (TFS). The RT-qPCR mix (100 μL) was loaded into custom TaqMan Advanced Human miRNA Custom Cards and assayed using a QuantStudio™ 7 Flex Real-Time PCR System. Each participant’s RT-qPCR data was imported into ExpressionSuite Software version 1.3 (TFS) for automatic baselining and thresholding, with maximum Cq set to 40 cycles, then exported from ExpressionSuite as a single file that included data from the 289 plasma samples and one water-and-no-RT control. Data was analyzed using Stata version 17.0 software.

### 2.5 MiRNA RT-qPCR quality control metrics

For each RT-qPCR well, we examined a variety of quality control (QC) metrics exported by ExpressionSuite software. Cq values for miRNA amplifications were included in statistical analysis if they had an AmpScore ≥1.0 with CqConf ≥0.8. MiRNA amplifications (e.g., let-7b-5p) that showed performance slightly below those thresholds (either AmpScore ≥0.9 with CqConf ≥0.8, or CqConf ≥0.75 with AmpScore ≥1.0) had Cq values accepted for statistical analysis conditional on meeting other criteria for good amplification (strong consistency of Cq values across triplicate wells). For accepted amplifications we examined the appropriateness of the baseline separation from the recorded Cq value and compared our QC assessments with other quality flags exported by QuantStudio, finding nothing unusual. Amplifications with acceptable QC but Cq > 35 were considered censored values and given special handling using censoring-aware methods in the analysis. Amplifications that did not meet the QC standards for accepted or censored amplifications were considered missing measurements, regardless of Cq value. Amplifications labeled “Undetermined” by QuantStudio were also considered missing measurements. As a final fidelity check we (1) examined the amplifications for the two negative control miRNAs (miR-1293 and miR-647) to verify that no sample had a Cq value recorded for either of those with acceptable QC, and (2) verified that the spike-in positive control (cel-miR-39-3p) and primary endogenous normalization control (miR-16-5p) were both completely observed with acceptable QC in all replicates for all samples. The other endogenous normalizer control miRNAs (miR-24-3p, miR-92a-3p, miR-204-5p) showed good performance, with expression of miR-24-3p and miR-92a-3p in 100% of samples and miR-204-5p in 77% of samples. Given our built-in redundancy of normalizer control miRNAs and model-based methods of normalization that can handle noninformative missing data, we considered this amount of normalizer measurement dropout acceptable.

### 2.6 Cq calibration and normalization

For technical calibration, all raw Cq values were adjusted for RNA isolation differences via median-alignment of the cel-miR-39-3p spike-in control reactions as an exogenous calibration factor. This adjustment represented the largest contribution to the overall normalization, with 57% of the technical variance explained by differences in spike-in control values (Supplementary Figure 1). (Note that we define technical variance as the variance in average Cq values across samples.) After technical calibration, all Cq values for the cel-miR-39-3p spike-in (being exactly aligned by median) were removed from the dataset and did not contribute to later normalization steps. Next, these technical calibrated Cq values were batch calibrated to remove residual artifacts attributed to processing order. We fit a linear mixed-effects regression model including the same 5-knot restricted cubic spline of the RT-qPCR run order as above, plus indicators for known abnormal sample or processing events (e.g., visible hemolysis), with PCR run batches entered as random intercepts. Fitted values from this regression were subtracted from the corresponding Cq values for all miRNAs as the adjustment for batch and order-related effects. Batch calibration adjustments were relatively minor for our dataset, accounting for 10% of the technical variance (Supplementary Figure 1).

Calibrated Cq values were then normalized using a mixed-effects model on the endogenous miRNA normalizer Cq values only. The model included quadratic effects of age (interaction effect of age with itself, to model the effect of age at differing ages rather than assuming the effect is linear for all ages), fully-interacted indicators for sex and diagnosis group to adjust out study factors (because we wish to retain these factors for analysis), and crossed random effects of miRNA and sample to model the miRNA-sample interaction. We used this model to estimate the residual differences between samples as BLUPs (best linear unbiased predictions). The BLUP values were subtracted from the fully calibrated Cq values as endogenous normalizer level effects across samples. The resulting normalized Cq values were recentered to the global median of the input Cq value distribution. This final normalization step accounted for 26% of the technical variance in the data (Supplementary Figure 1). The remaining 7% of technical variance was unexplained. After normalization, 83% of the total Cq variance (expected between-miRNA variance plus signal and residual technical and noise variance) was attributable to differences in mean expression across miRNAs (expected variance), 9% was attributable to sample-miRNA interactions (the biological signal), and <1% to differences in mean expression across samples (the residual technical variance, meaning the normalization was highly successful); the remainder of ~7% is residual variance (the measurement noise). We verified that the normalized Cq values of the normalizer miRNAs themselves had essentially flat profiles and noted that the normalization achieved substantial variance reduction.

### 2.7 Cq censoring and imputation

Censored Cq values are quantitatively unreliable because their measurement falls outside the linear range of the assay. These were treated as interval observations, defined as an unobserved value falling between an upper and a lower limit. For the purposes of regression on the Cq values as an outcome predicted by diagnosis status these can be handled as interval observations directly via modeling. However, when the Cq values are used as predictors of diagnosis status the interval observation must be collapsed to a single most-likely value falling within the interval. To accomplish this, we used a Bayesian multilevel interval regression model that considers all replicate wells for the sample jointly and naturally accounts for the different character of censored values in the context of non-censored values for the same miRNA. For the Bayesian prior distribution on the mean level for the miRNA, we used a Student’s *t* distribution with 4 degrees of freedom and a (wide and uninformative) scale of 10, and for the log of the root mean square error we used a (similarly uninformative) half-Cauchy distribution with the same scale of 10. Random effects for individual samples were assigned a zero-centered prior normal distribution with default conjugate inverse-gamma hyperprior (shape and scale parameters both 1/100) for the random-effect variance. The imputed value for a censored observation was taken as the posterior grand mean for the miRNA plus the posterior mean of the random effect, based on 10,000 Markov chain Monte Carlo draws from the posterior distribution, following a burn-in run of 2,500 draws. Note that the rank correlation between the predictions from this model and the naïve means of the corresponding replicate wells was 0.98, reflecting almost complete preservation of rank ordering among samples, but the model prediction is less biased because it accounts for censoring, which prevents it from overestimating the expression level. (The mean overestimate was 0.95 Cq.)

Prior to imputation the normalized Cq values, both censored and uncensored, are converted to an expression scale using inverted Cq units so that positive values mark higher expression. Non-censored Cq values with acceptable QC were used as exact measurements (both bounds of the interval are the same normalized Cq value). Censored Cq values with acceptable QC were treated as falling between the censoring threshold of 35 (lower bound) and the average (upper bound) of the normalized Cq value and the maximum normalized Cq value across all observations on all miRNAs (the “observed maximum,” a value that will be strictly less than the maximum cycle number of 40, specified as a machine setting). Missing Cq values were treated as falling between the observed maximum (lower bound) and the maximum cycle number (upper bound of 40). Non-missing Cq values with unacceptable QC were treated as missing measurements for most purposes, but in this context the recorded Cq (with all normalization adjustments) was used as the lower bound and the maximum cycle number (40) as the upper bound. Finally, all the interval bounds were subtracted from the maximum cycle number of 40 to invert the scale.

### 2.8 Statistical analysis of individual miRNAs

Individual miRNAs were assessed one by one for association with diagnosis group categories using maximum-likelihood estimation of a multilevel model specification. Models considered the miRNA expression value as an outcome predicted by diagnosis status, adjusted for age and sex, and used the Huber-White heteroskedasticity-robust standard error estimator, with a random effect of sample to account for correlation among the replicate wells for the same sample. For miRNAs having any censored values we adopted a multilevel interval regression model (that accounts for censored and missing Cq values) incorporating both the lower and upper bounds of expression for the censored values, and for miRNAs with no censored values we used an ordinary linear mixed-effects model instead. The statistics of interest were the coefficients on the diagnosis status terms, which represent the mean log_2_ miRNA expression associated with each diagnosis status, adjusted for age and sex. These coefficients were used to calculate the group contrast values for each miRNA based on the difference in values (i.e., log_2_ fold changes) for one diagnosis relative to another (e.g., AD vs. NC, AD vs. PD). A *z*-score for each group contrast was calculated by dividing the contrast value by the standard error of that difference as estimated by the model. For visualization purposes, the group contrast values were standardized to generate absolute contrast magnitudes and are depicted in heatmaps by the size of the boxes. The absolute contrast magnitude was calculated by dividing by the root-mean-square error and multiplying by the estimated intraclass correlation coefficient (ICC) from the multilevel model for the miRNA. The ICC is a reliability statistic that characterizes the average consistency of the triplicate wells for a given assay for a sample. A large ICC indicates that on average across samples the technical replicates for a given sample were very close in value thus there is higher confidence in the model average as a reliable estimate of the measured level for a sample, while a small ICC indicates that the technical replicates have large variance and thus give low confidence that the measured level is repeatable. For all of the miRNAs the ICC values were acceptably good (>0.6), and very good (>0.9) for most amplifications (Supplementary Table 2).

### 2.9 Statistical modeling of diagnosis classifiers

Prior to predictive modeling we generated an ‘AD-specificity’ score for each miRNA, which is based on the complete collection of group contrast values for a given miRNA and is used to qualitatively categorize that miRNA as more specific to AD, nonspecific, or more specific to PD and/or DLB. The score is proportional to the absolute value of the *z*-score for the AD-vs.-NC association with the miRNA (see Statistical Analysis of Individual MiRNAs above) multiplied by the square of the ratio of that same AD association z-score to the sum of the absolute z-scores for the PD-vs.-NC and DLB-vs.-NC associations with the miRNA:

$$\mathrm{AD}\;\text{specificity}\propto |z_{\mathrm{AD}}|\cdot {\left(\frac{z_{\mathrm{AD}}}{|z_{\mathrm{PD}}|+|z_{\mathrm{DLB}}|}\right)}^{2}$$

The interpretation of the score is that large values indicate that the miRNA expression changes substantially in AD where PD and DLB levels are close to those for NC, and conversely, small values indicate substantial expression changes for PD and/or DLB where AD levels are close to those for NC. We scaled this score so that the value would be near 1 (i.e., near 0 on the log scale) when the relative difference between the AD association z-score and the mean of the PD and DLB association z-scores was not larger than ~1.4 (the square root of 2, the expected standard deviation of differences between two *z*-scores), and classified the set of miRNAs into three classes according to whether the score was greater than ~4 (specific to AD), less than ~1/4 (specific to PD and/or DLB), or in between those two cutoff values (nonspecific); this comparison was done on the log scale for additive symmetry. Larger positive log scores represent greater AD specificity, and larger negative log scores greater PD or DLB specificity (Supplementary Table 3).

We next applied prediction modeling strategies to assess the performance of AD classifiers on discrimination of the non-AD dementias PD and DLB, with the goal of ascertaining the feasibility of an AD classifier that was both sensitive (discriminating AD from NC) and specific (selecting AD within the context of the other dementias). Our testing plan involved the fitting and characterization of four distinct AD classification models that we specified *a priori*:

1. “Empirical” model — elastic-net logistic regression (ENR) model for AD vs. pooled {PD + DLB}, considering all 57 miRNAs, adjusted for age and sex, with mixing and penalty parameters tuned so that all 57 miRNAs would only just be included (i.e., set so that a slight change in either tuning parameter would remove one or more of the miRNAs).
2. “AD-specific” model — ridge logistic regression model for AD vs. pooled {NC + PD + DLB} comprising only the 10 miRNAs identified as AD-specific according to our score cutoff (see Statistical Modeling of Diagnosis Classifiers above), adjusted for age and sex.
3. “Top-2” model — unadjusted ordinary logistic regression model for AD vs. pooled {PD + DLB} containing our “top-2” (log) miRNA ratio proportional to miR-23a-3p expression divided by miR-423-5p expression, as proposed in a prior study (Sandau et al., 2025) as the sole predictor.
4. “Big effects only” model — ridge logistic regression model for AD vs. NC (only), using the 4 miRNAs (miR-143-3p, miR-145-5p, miR-34a-5p, and miR-584-5p) that demonstrated the largest absolute magnitudes of effect for AD vs. NC in individual-miRNA models, adjusted for age and sex; the number 4 was chosen a priori based on past experience with prediction modeling in this context, and the motivation for this model was to explore how well other non-AD dementia groups could also be discriminated when using a classifier naively built based on information comparing AD to NC absent the context of those other dementias.

After each model was fit, we generated predicted probabilities of AD given the model and assessed the classification performance of those predicted probabilities for each of the disease groups (AD, PD, DLB) vs. NC and AD vs. the other dementias, both individually and pooled, by calculating the 7-fold cross-validated area under the receiver operating characteristic (ROC) curve (cvAUC) and the standard deviation (SD) of the cvAUC. Bootstrap bias-corrected 95% confidence intervals (CIs) for cvAUC were also calculated and are reported in square brackets along with the cvAUC values below. We used the same 7 folds for cross-validating the performance of all prediction models so that the same subgroupings of observations were being interrogated in the same way for all models under consideration, ensuring direct comparability of performance across models.

Finally, we visualized the distinctiveness of the disease groups only (i.e., not NC) with respect to the 57 miRNAs by performing a linear discriminant (LD) analysis employing proportional prior probabilities for diagnosis group membership and leave-one-out cross-validation of the functional parameter estimation. The cvAUC and SD of cvAUC for each boundary line (treated as a classification rule) were calculated, along with bootstrap bias-corrected CIs. The relative importance of each miRNA’s contribution for defining the coordinates of this space are captured by the individual loadings (signed weights ranging from −1 to 1) of the miRNA in the additive combination defining each of the two discriminant functions; we highlighted miRNAs as specific to a dimension if their loading was larger in magnitude than 0.7 on one discriminant function and smaller than 0.3 on the other discriminant function. We used this analysis to characterize the overall degree of miRNA expression differences among the disease groups, but it is important to note that it does not directly address the diagnostic potential of the miRNAs (only their distinctiveness across the dementias) because the distribution of the NC population within the space was not considered. To measure overall diagnostic potential, we calculated distances between each pair of observations using a random forest classifier of the diagnosis groups with all 57 miRNAs as predictors, then performed a nonmetric multidimensional scaling (MDS) of the distances to obtain a two-dimensional projection of the miRNA expression information that preserves the proximity ranking (Supplementary Figure 2). Lines of best separation for each group vs. all the others in this MDS space were calculated via logistic regressions on the MDS coordinates, and cvAUC and SD of cvAUC were also calculated.

### 2.10 MiRNA target prediction and pathway analysis

We used TargetScan 7.2 and miRDB to predict targets for miRNAs of interest. As pathway analysis is most effective for predictions generated from a limited gene set, predicted targets were excluded if they had a Cumulative Weighted Context Score (CWCS) > −0.3 in TargetScan or a target score of <80 in miRDB. Pathway analysis was then performed using Ingenuity Pathway Analysis (IPA; QIAGEN Inc) software, excluding cancer-related tissues and cell lines to avoid knowledge bias towards cancer.

## 3 Results

### 3.1 Participant demographics and clinical characteristics

Plasma samples from 289 participants included 82 NC, 87 AD, 100 PD, and 20 DLB were used for miRNA analysis (Table 1). Study variables on participants included age at time of plasma collection, self-reported sex, and cognitive scores. Considering that groups were not balanced with respect to either age, *p* < 0.001, or sex, *p* = 0.011, all analyses were adjusted for these factors (Table 1). The NC and AD participants had cognitive assessment via the MMSE while the PD and DLB participants were assessed by the MoCA. The raw MoCA scores for PD and DLB participants were converted to an equivalent weighted MMSE score to enable comparison with AD and NC participants (Fasnacht et al., 2023) (Table 1; Supplementary Figure 3). As expected, there were large group differences between AD and DLB vs. NC and PD but not between AD vs. DLB or PD vs. NC (Table 1). The vast majority of NC and PD participants had MMSE scores >25, while the majority of AD participants had MMSE <25 (Supplementary Figure 3). The DLB participants had a bimodal distribution of MMSE scores with ~50% of the DLB participants overlapping with AD participant scores and the remaining DLB participants showing a similar distribution to PD and NC participants (Supplementary Figure 3).

### 3.2 Specificity of 57 AD-associated MiRNAs for AD, PD, and DLB

**Figure 2:**
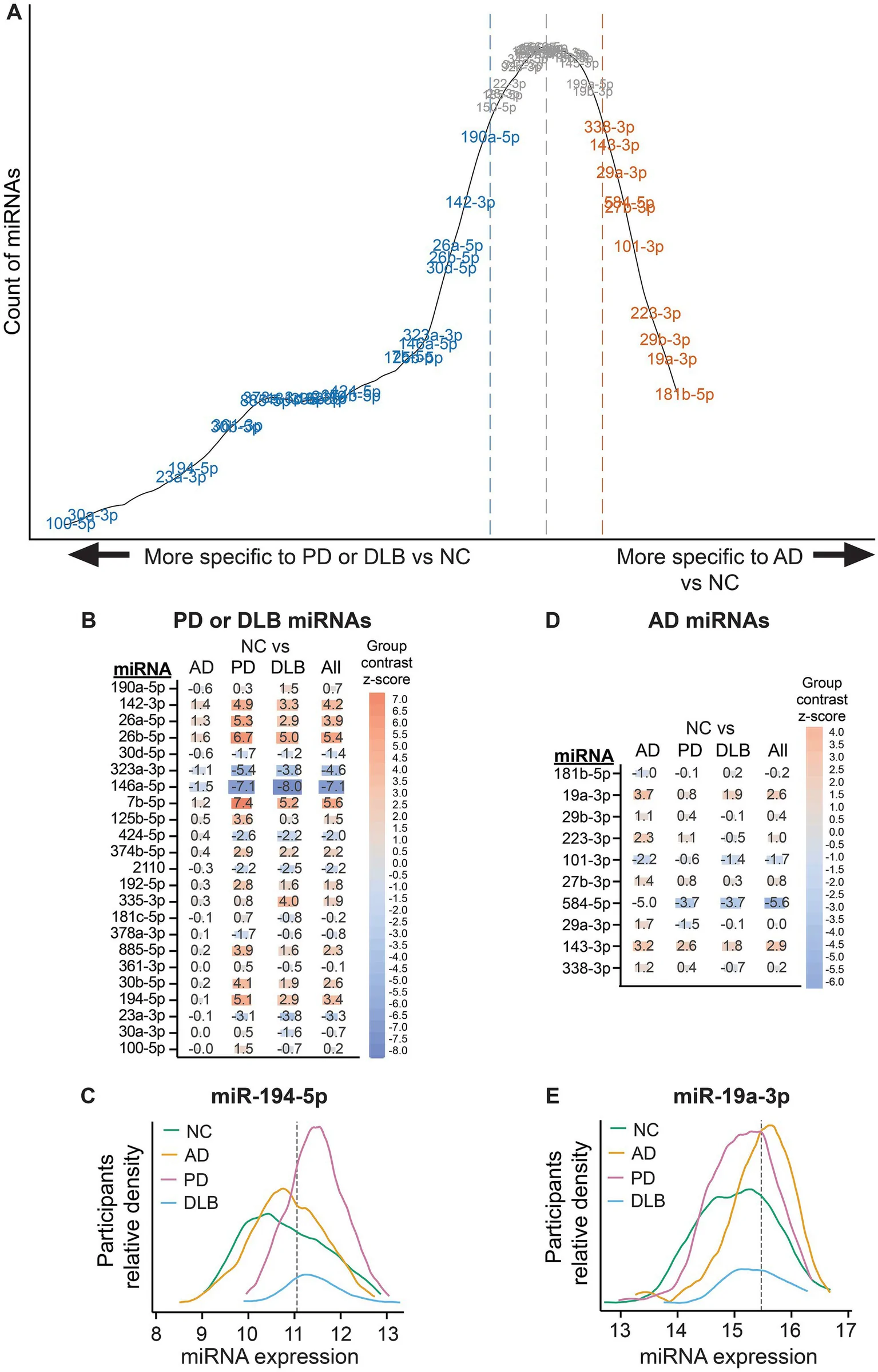
Categorization of miRNAs as more specific to AD or more specific to PD and/or DLB. **(A)** Distribution of the 57 miRNAs along the scale of an AD-specificity score based on comparison of group contrast values (NC vs. AD, NC vs. PD, NC vs. DLB) for each miRNA. MiRNAs were qualitatively categorized as more specific to PD or DLB (blue: 23 miRNAs), nonspecific to disease state (gray: 24 miRNAs), or more specific to AD (orange: 10 miRNAs) based on the log scaled AD-specificity scores. **(B)** Group contrasts of the miRNAs for NC vs. AD, NC vs. PD, NC vs. DLB, and NC vs. All {AD+PD + DLB} for the 23 PD- and/or DLB-specific miRNAs. **(C)** Relative density plot showing the distribution of participants within the NC (green), AD (yellow), PD (pink), and DLB (blue) groups for miR-194-5p expression levels, a representative PD- and/or DLB-specific miRNA. **(D)** Group contrasts of the miRNAs for NC vs. AD, NC vs. PD, NC vs. DLB, and NC vs. All {AD+PD + DLB} for the 10 AD-specific miRNAs. **(E)** Relative density plot showing the distribution of participants within the NC (green), AD (yellow), PD (pink), and DLB (blue) groups for miR-19a-3p expression levels, a representative AD-specific miRNA. **(B,D)** The heatmap cell values, hue (negative = blue, positive = red), and color intensity reflect the z-score of the corresponding contrast from the miRNA expression association models. The cell size is proportional to the standardized absolute contrast magnitude. **(C,E)** Vertical dashed lines represent the cut point for miRNA expression levels where the higher density of AD and NC participants were on opposite sides.

In our prior study, we used machine learning to identify multi-miRNA models based on plasma expression levels for 57 candidate AD miRNAs to classify AD from NC (Sandau et al., 2025). Recognizing the limitation that this binary comparison may identify non-specific “neurodegeneration miRNAs,” we sought to determine the specificity of those 57 miRNAs for AD vs. PD and/or DLB. We first assessed the miRNAs individually by calculating an AD-specificity score for each miRNA, which is a composite score that incorporates the three disease groups vs. NC comparisons (*z*-scores for AD vs. NC, PD vs. NC, DLB vs. NC) into a single metric. Using the AD-specificity score we qualitatively categorized each miRNA as (1) more specific to PD or DLB, (2) more specific to AD, or (3) distinct from NCs but nonspecific to disease state (Figure 2A; Supplementary Table 3). Based on the AD-specificity score, 23 of the 57 miRNAs were categorized as more specific to PD or DLB (Figure 2A, blue), 10 miRNAs were more specific to AD (Figure 2A, orange), and 24 miRNAs were not specific to disease state (Figure 2A, gray). Since the AD-specificity score provides minimal information regarding the impact of each disease state individually, we generated heatmaps of the miRNA associations to visualize the magnitude of change (cell size), degree of significance (color intensity), and direction of change (cell hue) for AD vs. NC, PD vs. NC, and DLB vs. NC (Figures 2B,D; Supplementary Figure 4A). Within the miRNAs categorized as more specific to PD or DLB, miR-194-5p was ranked fourth vs. NC (Figures 2A,B) with density plots showing marked increased expression in PD (pink) vs. NC (green) that was slightly attenuated in DLB (blue) vs. NC (green), and negligible expression differences in AD (yellow) vs. NC (green) (Figure 2C). A heatmap for the 10 AD-specific miRNAs shows ones where changes in expression were generally greatest in AD vs. NC, as opposed to PD vs. NC and/or DLB vs. NC (Figure 2D). However, for the AD-specific miRNAs the group differences in the magnitude of change and degree of significance (Figure 2D) were generally not as large as PD/DLB-specific miRNAs (Figure 2B). Within the 10 AD-specific miRNAs, miR-19a-3p showed increased expression in AD vs. NC, while DLB and PD generally overlapped with NC (Figure 2E). For the 24 miRNAs categorized as nonspecific to disease (Supplementary Figure 4A), ~75% of the miRNAs had altered expression levels in AD, PD, and/or DLB vs. NC and thus may serve as general biomarkers for neurodegenerative disease; an example is miR-423-5p (Supplementary Figure 4B). The remaining miRNAs, such as miR-660-5p, had comparable expression levels between all groups including NC (Supplementary Figure 4C). Together, these data demonstrate that plasma expression levels of some miRNAs are altered by neurodegeneration in general while others are disease-specific.

### 3.3 Sex-dependent effects on plasma miRNA expression in AD, PD, and DLB

**Figure 3:**
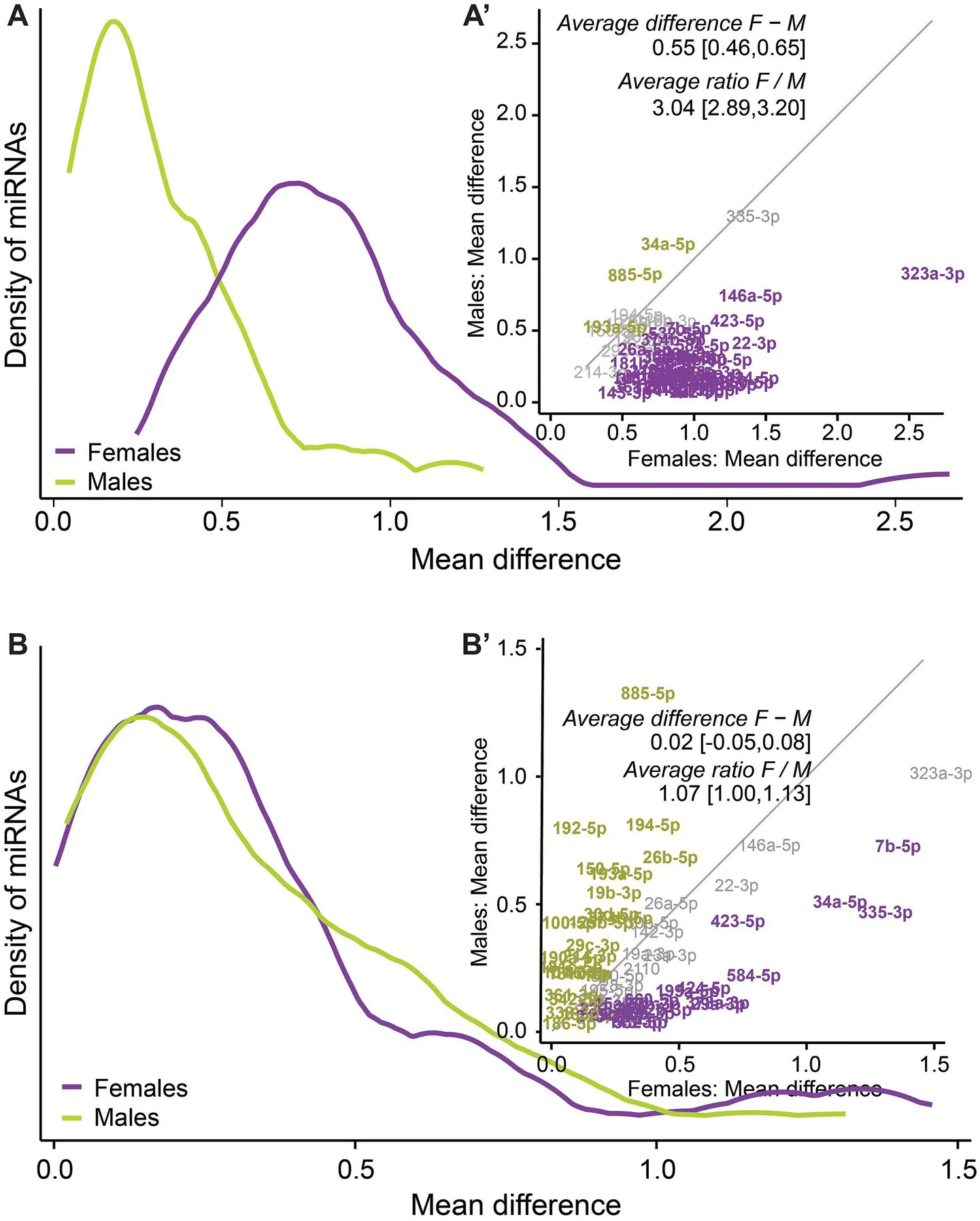
Sex-dependent differences in plasma miRNA expression among AD, PD, and DLB participants. **(A)** Age-adjusted mean absolute differences in expression among AD, PD, and DLB groups for each miRNA were calculated separately for the female and male subcohorts. The distributions of mean absolute differences showed similar right skew for both sexes, but the distribution for females (purple) had larger skewness and a significantly higher central location compared to the distribution for males (yellow-green). **(A′)** Comparison plot of females and males on the 57 miRNAs based on their mean difference. Note that the average mean difference across all miRNAs was ~3x larger for females (average difference F–M = 0.55 Cq). MiRNAs were color-coded as more robust in males (yellow-green), neutral (gray), or more robust in females (purple) based on the ratio of the mean difference (female/male) for each miRNA. The cutoff was set as neutral for ratios between 2/3 and 3/2 and otherwise set as sex-specific. That implies an absolute log_2_-ratio of 0.585 as the cutoff. **(B,B′)** Sensitivity analysis showing the distributions and comparisons of female and male mean absolute differences in expression between AD and PD groups for each miRNA, omitting DLB participants entirely. The dramatic attenuation in the mean female/male difference and ratio suggests that the pattern of systematically larger female effect sizes may not be generalizable, or may be a DLB-specific phenomenon.

Considering that there are sex-differences in both AD and PD with increased incidence of AD in women (Sandau et al., 2025) as opposed to increased incidence of PD in males (Willis et al., 2022) and considering our prior publication identifying sex as a potential moderator of AD classification accuracy using plasma multi-miRNA models (Sandau et al., 2025), we assessed the impact of sex on plasma miRNA expression in our AD, PD, and DLB samples. However, it is important to note that this analysis is exploratory since sample availability constraints resulted in sex bias with a greater proportion of males in all groups, especially the DLB group. For this analysis we calculated age-adjusted mean absolute differences in expression among AD, PD, and DLB groups for each miRNA for the female and male subcohorts. We found that for females most miRNAs had a significantly larger mean absolute difference in expression (purple) compared to males (green-yellow) (Figure 3A). Out of the 57 miRNAs, 44 had more robust expression differences in AD, PD, and/or DLB females (purple), compared to only 3 that were more robust in males (green-yellow) and 10 that were neutral (gray) (Figure 3A; Supplementary Table 4). We further illustrate this in a participant relative density plot for miR-19a-3p, which is a representative miRNA where diagnosis group differences were ~3x larger for females than for males (Supplementary Figure 5A). In females, there were more defined group separations based on miR-19a-3p expression levels, with low to high expression generally associated with NC compared to PD and still higher expression for AD; conversely, in males miR-19a-3p expression levels tended to be more similar between the groups (Supplementary Figure 5A). Analogously, miR-885-5p was one of three miRNAs where diagnosis group differences were ~3x larger for males than females, and we see more defined group separations in males compared to females (Supplementary Figure 5B). However, a severe limitation of this analysis is that the disease-specific male–female contrasts each rely on sex-biased samples, and in the case of DLB an extremely biased and size-limited sample (only 2 females out of 20 total DLB examples). As a sensitivity analysis, we excluded the DLB group entirely from consideration and recalculated the age-adjusted mean absolute differences in expression for the male and female subcohorts among AD and PD groups only (Figure 3B). Although the AD and PD samples represent ~90% of the diseased (non-NC) cohort, the results under this sensitivity analysis were dramatically attenuated (mean female:male ratio of just 1.07 vs. 3.04; Figures 3A,B) compared to when the DLB samples were also included, suggesting that the systematically larger group differences we observed in females relative to males were largely driven by evidence from the tiny number of DLB females, and may not be a robust result if the DLB evidence is unrepresentative. An alternative interpretation is that the pattern of larger female group separations is a DLB-specific phenomenon, where DLB females differ much more from AD and PD females than their male counterparts do in terms of plasma miRNA expression. While these results are based on an exploratory analysis with questionable generalizability, they raise the possibility that in neurodegenerative disease there may be more robust changes in plasma miRNA levels in females compared to males.

### 3.4 Classification performance for AD vs. PD and/or DLB using AD-specific miRNAs

**Figure 4:**
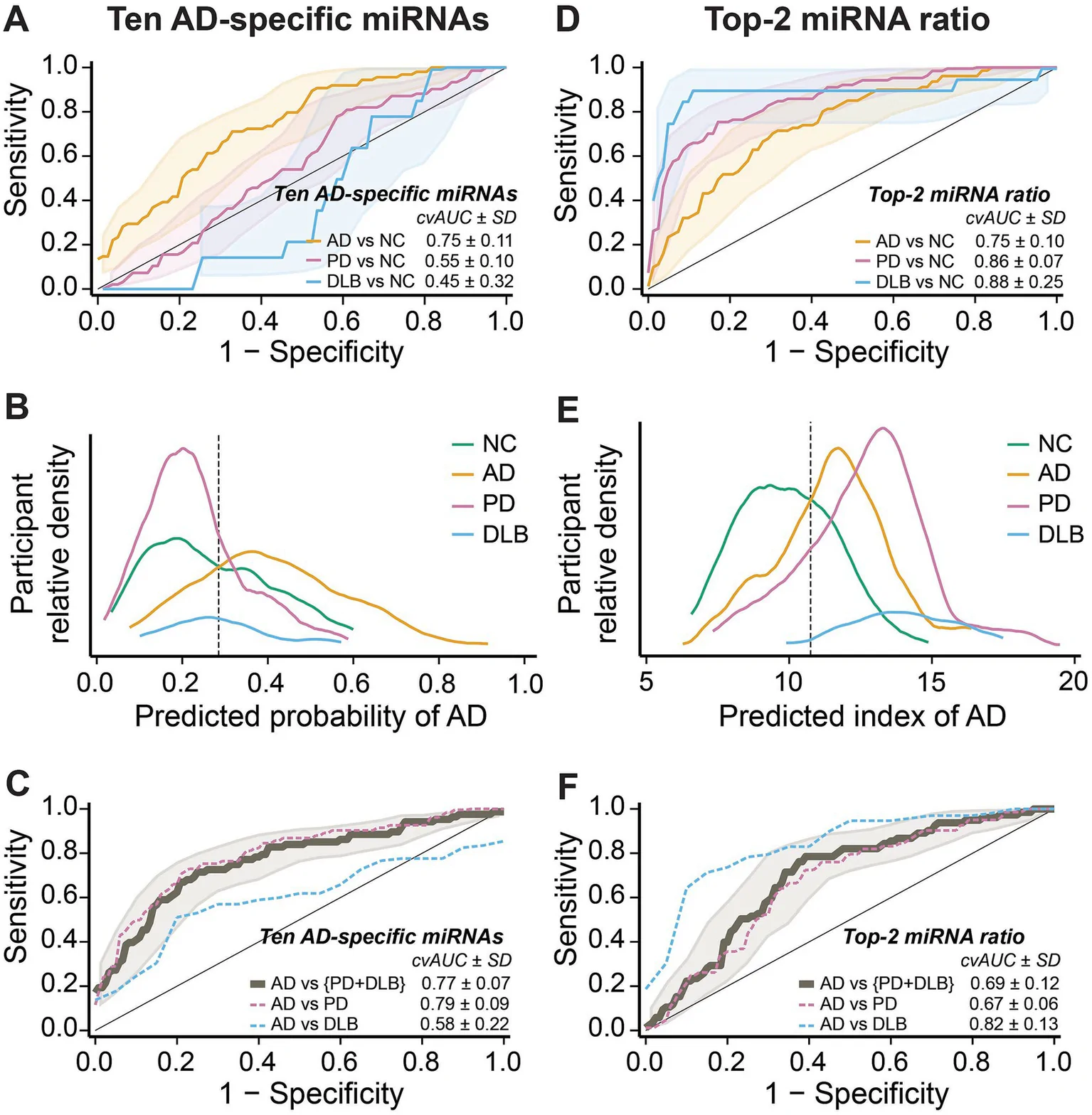
Classification of AD from PD and DLB using AD-specific multi-miRNA models. **(A–C)** Characteristics of a ridge logistic regression model for AD vs. {NC + PD + DLB} (pooled) comprising only the 10 miRNAs identified as AD-specific according to our score cutoff and adjusted for age and sex. **(D–F)** Characteristics of an (unadjusted) ordinary logistic regression model for AD vs. {PD + DLB} containing our “top-2” (log) miRNA ratio proportional to miR-23a-3p expression divided by miR-423-5p expression, as proposed in a prior study (Sandau et al., 2025) as the sole predictor. **(A,D)** Receiver operating characteristic (ROC) curves for each of the disease groups (AD, PD, DLB) vs. NC using the model-derived predicted probability for the **(A)** AD-specific miRNAs or **(D)** top-2 miRNA ratio as the group classifier. Mean area under the ROC curve (cvAUC) and standard deviation (SD) of cvAUC were calculated by 7-fold cross-validation, using the same 7-fold divisions for all group comparisons. **(B,E)** Relative density plots showing model-derived predicted probabilities **(B)** or the AD index **(E)** for participants within the NC (green), AD (yellow), PD (pink), and DLB (blue) groups for the **(B)** AD-specific miRNAs and **(E)** top-2 miRNA ratio. **(C,F)** ROC curves for AD vs. PD, DLB, and {PD + DLB) (pooled) using the model-derived predicted probability for the **(C)** AD-specific miRNAs or **(F)** top-2 miRNA ratio as the group classifier. cvAUC ± SD calculated by 7-fold cross-validation as above.

We next assessed the performance of the 10 AD-specific miRNAs (Figure 2D) when combined into a single classifier for AD vs. PD and/or DLB (Figure 4A). As expected, this AD-specific miRNA classifier achieved good separation of AD from NC (cvAUC = 0.75 [0.66, 0.82]) and almost no separation of either PD (cvAUC = 0.55 [0.46, 0.62]) or DLB (cvAUC = 0.45 [0.35, 0.54]) from NC (Figure 4A). This is further illustrated with a participant relative density plot that shows a majority of the AD participants had a higher predictive probability of AD (orange) compared to the PD (pink) and DLB (blue), which generally had a lower probability of being AD and more overlap with NC (green), although AD was less distinct from DLB than from PD (Figure 4B). In line with this, the AD-specific miRNA classifier had good performance for separating AD vs. PD and DLB (cvAUC = 0.77 [0.70, 0.83]) and AD vs. PD (cvAUC = 0.79 [0.71, 0.85]), but not as much for AD vs. DLB (cvAUC = 0.58 [0.43, 0.79]) (Figure 4C). As another approach, we combined the plasma expression data for the 4 miRNAs with largest effect size for AD vs. NC (miR-143-3p, 145-5p, 34a-5p, 584-5p) into a single classifier and found that while there was good separation for AD, PD, and DLB vs. NC (Supplementary Figure 6A), together these miRNAs are unable to reliably classify AD from PD and/or DLB (Supplementary Figures 6B,C). We also assessed the performance of our previously-identified miRNA classifier based on the ratio of two miRNAs (miR-23a-3p and 423–5) that had similar performance for classifying AD vs. NC compared to models combining information from 57 miRNAs (Sandau et al., 2025). Here we recapitulated the performance of the top-2 miRNA classifier for AD vs. NC (cvAUC = 0.75 [0.66, 0.82]; Figure 4D), just as we saw in our prior study (Sandau et al., 2025). But interestingly, the top-2 miRNA classifier showed even larger signal for both PD (cvAUC = 0.86 [0.79, 0.92]) and DLB (cvAUC = 0.88 [0.76, 0.97]) vs. NC (Figure 4D). This finding reinforces that the miRNA assay captures generic information relative to neurodegeneration, and that studies to identify specific miRNAs associated with distinct dementias are needed. This is further illustrated in the relative density plot that shows a majority of both PD (pink) and DLB (blue) had a greater separation from NC (green) compared to AD (orange; Figure 4E). These results underscore the need to consider broader dementia contexts to maintain disease specificity when identifying and proposing diagnostic biomarkers.

### 3.5 Robust classification performance for AD vs. PD and/or DLB using elastic-net regression predictive modeling

**Figure 5:**
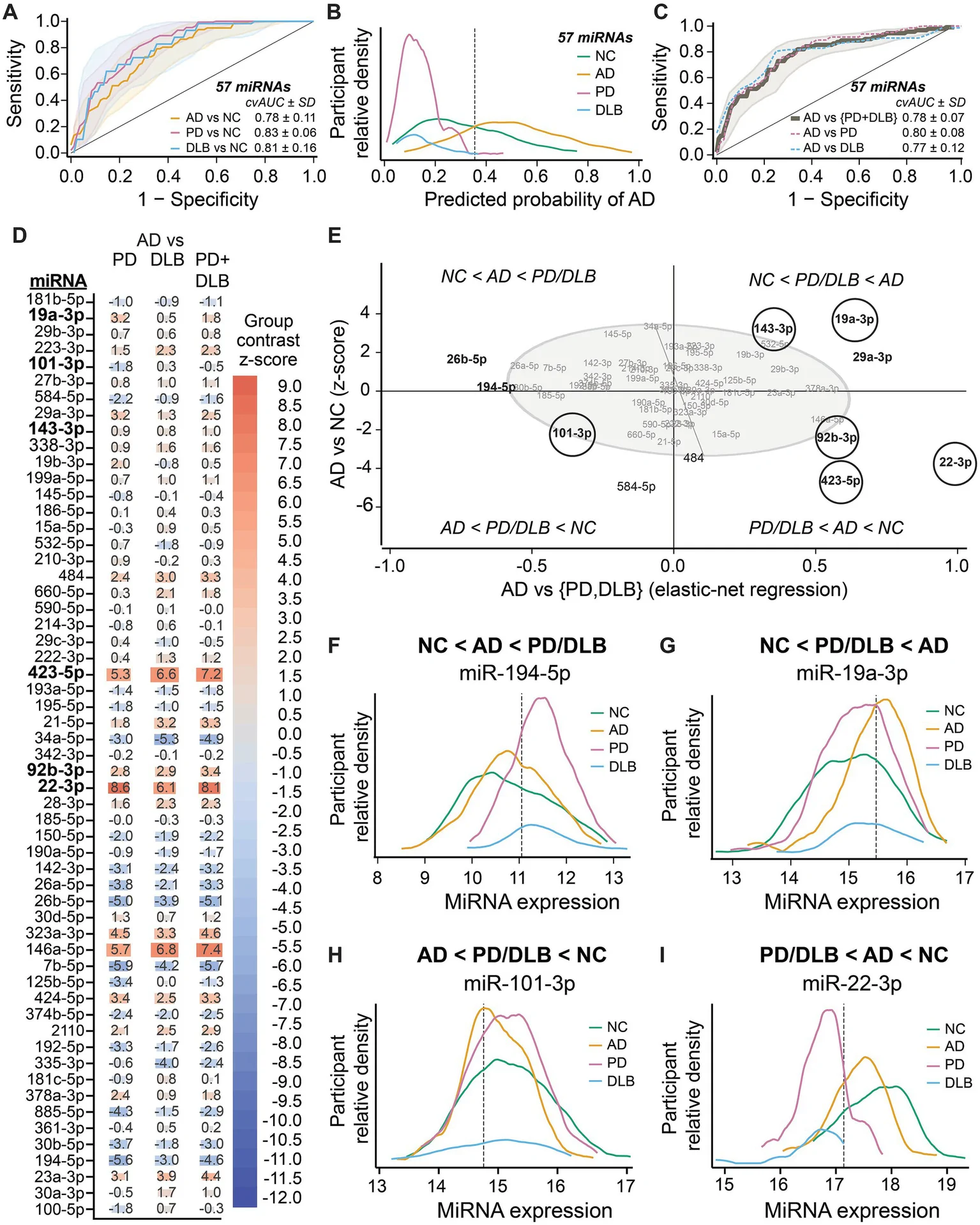
Six miRNAs contribute the most to a multi-miRNA classification model for AD vs. PD and/or DLB. **(A–C)** Characteristics of an EN logistic regression model for AD vs. {PD + DLB} (pooled), considering all 57 miRNAs and adjusted for age and sex. **(A)** Receiver operating characteristic (ROC) curves for each of the disease groups (AD, PD, DLB) vs. NC using the corresponding model-derived predicted probability as the group classifier. Mean area under the ROC curve (cvAUC) and standard deviation (SD) of cvAUC were calculated by 7-fold cross-validation, using the same 7-fold divisions for all group comparisons. **(B)** Relative density plot showing the distributions of participants based on the model-derived predicted probabilities for NC (green), AD (yellow), PD (pink), and DLB (blue) groups. The vertical dashed line represents the cutpoint for predicted probability of AD vs. NC. **(C)** ROC curves for AD vs. PD, DLB, and {PD + DLB} (pooled) using the corresponding model-derived predicted probability as the group classifier, with cvAUC ± SD calculated by 7-fold cross-validation as above. **(D)** Group contrasts AD vs. PD, AD vs. DLB, and AD vs. PD + DLB sorted according to the AD-specificity score. Cell values, hue (negative = blue, positive = red), and color intensity reflect the z-score of the corresponding contrast from the miRNA expression association models. Cell size is proportional to the standardized absolute contrast magnitude. **(E)** Top miRNAs contributing to the classification of AD vs. PD and/or DLB. Background scatterplot shows the coordinates of each miRNA with respect to separation of AD from NC (vertical axis, z-score of AD association coefficient) and AD from PD and/or DLB (horizontal axis, signed miRNA importance from ENR classifier for AD vs. pooled {PD + DLB}). Six circled miRNAs identified as distinct in AD vs. NC and vs. both PD and DLB. The shaded oval represents the expected scatter (in two dimensions) of the coordinates under Mahalanobis scaling. **(F–I)** Relative density plots showing the distribution of participants within the NC (green), AD (yellow), PD (pink), and DLB (blue) groups for the expression levels of a representative miRNA from each quadrant of the scatterplot in panel **(E)**. Vertical dashed lines represent the cutpoints for miRNA expression levels where the higher density of AD and NC participants were on opposite sides. Note that miRNAs in **(G)** upper-right (miR-19a-3p) and **(H)** lower-left (miR-101-3p) quadrants were AD-specific with AD (orange) distinct from NC (green), PD (pink), and DLB (blue) while **(F)** upper-left (miR-194-5p) and **(I)** lower-right (miR-22-3p) miRNAs showed intermediate expression of AD between NC and the combined PD and DLB groups.

In our prior study, we employed ENR to identify robust AD prediction models using plasma expression levels of the same 57 miRNAs examined herein (Sandau et al., 2025). Thus, we sought to determine if this machine-learning method with data for all 57 miRNAs in NC, AD, PD, and DLB participants would generate a robust multi-miRNA classifier for AD vs. PD and/or DLB. The ENR models incorporating all 57 miRNAs achieved roughly equal separation of NC (green) from AD (orange, cvAUC = 0.78 [0.69, 0.84]), PD (pink, cvAUC = 0.83 [0.76, 0.88]), and DLB (blue, cvAUC = 0.81 [0.69, 0.89]; Figure 5A). Importantly, while the cvAUC of ~0.8 for all disease groups vs. NC is similar (Figure 5A), the 57-miRNA model predicted that a majority of the NC, PD, and DLB participants had a very low probability of AD diagnosis, whereas most of the AD participants had a moderate to high predictive probability of AD (Figure 5B). In line with this, the classification performance of the 57-miRNA model showed good separation of AD (orange) from PD (pink, cvAUC = 0.80 [0.72, 0.86]) and DLB (blue, cvAUC = 0.77 [0.64, 0.87]), as well as the pooled group of PD and DLB samples ({PD + DLB} gray, cvAUC = 0.78 [0.71, 0.83]) (Figure 5C). This result suggests that the full set of miRNAs contains sufficient information to both sensitively and specifically select AD or PD/DLB patients from neurologically normal controls.

We next sought to determine the subset of miRNAs that contributed most to the classification performance of the ENR all 57-miRNA models. To visualize differences in miRNA expression between AD and the other disease groups, we generated a heatmap for the 57 miRNAs where the cell size represents the magnitude of change, color intensity is degree of significance (*z*-score), and cell hue shows the direction of change (Figure 5D). Generally, for each miRNA the direction and magnitude of change was similar between AD vs. PD and AD vs. DLB (Figure 5D). For instance, miR-146a-5p shows a large increase in expression in both PD and DLB vs. AD (Figure 5D). However, a limited number of miRNAs had differential expression in AD vs. PD but not AD vs. DLB (miR-125b-5p) or vice versa (miR-335-5p) (Figure 5D). To identify the top miRNAs contributing to the classification of AD vs. PD and/or DLB, all 57 miRNAs were plotted based on their separation of AD from NC (vertical axis) in relationship to their performance discriminating AD from PD and/or DLB (horizontal axis). The six circled miRNAs (miR-19a-3p, 22-3p, 92b-3p, 101-3p, 143-3p, 423–5p) were identified as distinct for AD vs. NC as well as distinct for AD vs. both PD and DLB. Note that only the upper-right and lower-left quadrants represent miRNAs that we call “AD-specific” in the sense that AD (orange) is distinct from the combined NC (green), PD (pink), and DLB (blue), i.e., where AD shows the most extreme miRNA expression. For example, the expression levels for miR-19a-3p had increasing levels of expression from NC to PD/DLB to AD participants (Figure 5G) while miR-101-3p had lowest expression levels in AD (Figure 5H). The upper-left and lower-right quadrants represent miRNAs where AD is different from the other groups but in the sense of having intermediate expression between NC and the combined PD and DLB, i.e., where PD and/or DLB are most extreme. For example, miR-194-5p showed an ordered increase in expression level from NC to AD to PD/DLB (Figure 5F) while miR-22-3p showed increasing levels of expression from PD/DLB to AD to NC (Figure 5I). A model consisting of only the 6 top-contributing miRNAs (Figure 5E: miR-19a-3p, 22-3p, 92b-3p, 101-3p, 143-3p, 423–5p) was found to achieve similar performance (AUC ~ 0.8) to the 57-miRNA model albeit with higher variance (data not shown). This suggests that classification with the 57-miRNA model was predominantly dependent on information from 6 miRNAs and that the other 51 miRNAs improve precision (in the sense of reducing residual errors in predicted probabilities) but not accuracy. Ingenuity Pathway Analysis (IPA) with these 6 miRNAs identified that within the Neurological Disease category the top 25 diseases and functions likely to be impacted by the mRNA targets include cognitive impairment, progressive neurological disorder, and movement disorder (Supplementary Table 5). Also, the most significant canonical pathway for the predicted targets of these 6 miRNAs was the Synaptogenesis Signaling pathway with predicted mRNA targets including ionotropic AMPA receptor subunit 1 (*GRIA1*), Synapsin-1 (*SYN1*), Synapsin-2 (*SYN2*), and multiple Synaptotagmin (*SYT1, SYT3, SYT6, SYT10, SYT11*) genes (Supplementary Table 6).

### 3.6 Individual participant discrimination by disease using plasma multi-miRNA models

**Figure 6:**
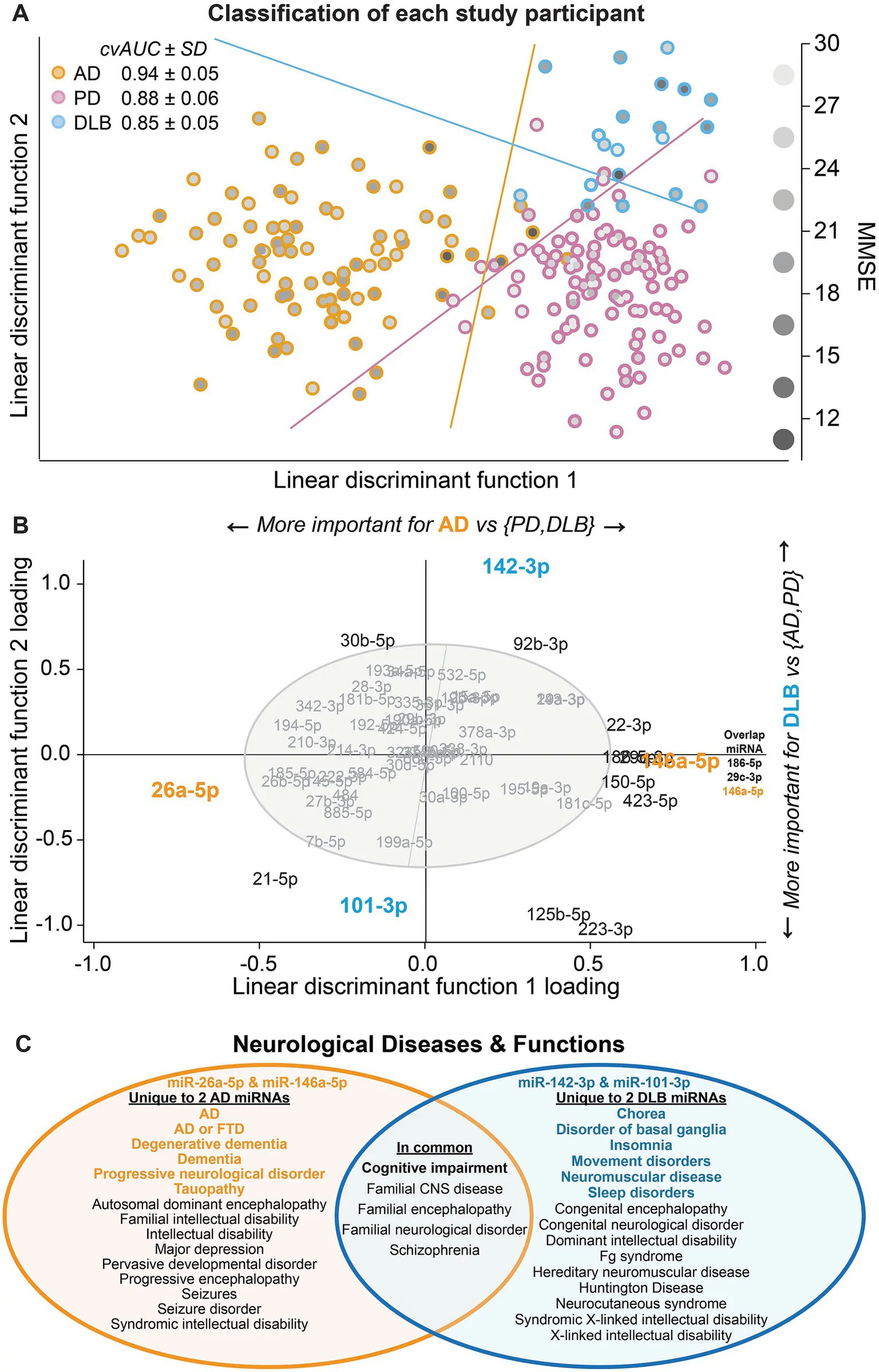
Separation of AD, PD, and DLB participants in relationship to cognitive status using the 57 miRNA model. **(A)** Classification of each study participant by LD analysis using proportional prior probabilities for diagnosis group membership and leave-one-out cross-validation of the functional parameter estimation was used to generate the two-dimensional functional space through which straight lines were drawn to optimally separate the groups within the space. Mean area under the receiver operating characteristic curve (cvAUC) and standard deviation (SD) of cvAUC were calculated by 7-fold cross-validation, using the same 7-fold divisions for all group comparisons. Mini-Mental State Examination (MMSE) scores for all participants were represented as shades of gray (MMSE>27 light gray to MMSE<12 dark gray) and used to indicate cognitive status (circle interior color). **(B)** Loadings for all 57 miRNAs on the two LD functions from the analysis in panel A. Larger absolute value of loading indicates a more important contribution from the miRNA for defining the discriminant function. The shaded oval represents the expected scatter (in two dimensions) of the function coordinates under Mahalanobis scaling. Those miRNAs falling outside of that oval are shown in larger font. The horizontal axis (first LD function) approximately divides AD (orange) from PD (pink) and DLB (blue) in the space, indicating importance for discriminating AD. The vertical axis (second LD function) approximately divides DLB from AD and PD in the space, indicating importance for discriminating DLB. Highlighted miRNAs showed greatest discrimination of AD (orange, large function-1 loadings and small function-2 loadings) and DLB (blue, large function-2 loadings and small function-1 loadings). **(C)** Venn diagram of the top 20 diseases or functions in IPA’s Neurological Disease category with a significant overrepresentation of the two miRNAs most important for discriminating AD vs. PD and DLB (miR-26a-5p, miR-146a-5p) and the analogous two for DLB vs. AD and PD (miR-142-3p, miR-101-3p). Diseases and functions in bold orange or blue font are unique to the AD or DLB miRNAs and clinically relevant to AD or DLB/PD, respectively.

As an orthogonal machine-learning approach to our ENR analysis (Figure 5), we used LD analysis with data for all 57 miRNAs to identify a two-dimensional multi-miRNA model that best separates AD from PD + DLB participants (LD function 1) and DLB from AD+PD participants (LD function 2) (Figure 6A). LD analysis is a supervised machine-learning approach to maximizing multivariate distance between groups in a low-dimensional space while minimizing the within-group variance. The LD functions achieved very good classification of AD vs. PD + DLB (cvAUC = 0.94 [0.87, 0.97]) with only 5 participants (orange circles) being classified to the right of the orange boundary that optimally separates the AD from PD + DLB groups (Figure 6A). Likewise, there was a very good separation of PD from AD+DLB (cvAUC = 0.88 [0.80, 0.92]) and DLB from AD+PD (cvAUC = 0.85 [0.78, 0.89]) with 8 PD participants (pink circles) being classified to the left of the pink boundary and 3 DLB (blue circles) below the blue boundary (Figure 6A). Considering that a small percentage of the participants from each group had overlapping MMSE scores (Supplementary Figure 3), we sought to determine if participant cognitive status impacted the classification. For example, were the AD participants with the highest MMSE scores classified to the right of the orange axis. MMSE scores were represented as shades of gray (MMSE>27 light gray to MMSE<12 dark gray) and used to indicate each participant’s cognitive status based on the inner circle shading. Generally, there was not a clear association between MMSE score and participant classification for any group. For example, two of the five AD participants that classified adjacent to the orange axis had severe cognitive impairment (dark gray = MMSE<15) (Figure 6A). Furthermore, there was a wide range in MMSE scores (light gray to gray = MMSE ~27–12) for DLB participants that were the greatest distance above the blue axis (Figure 6A). We also assessed the potential impact of residual age differences between particularly PD and AD (Table 1) by performing a sensitivity analysis repeating the LD analysis using propensity-score weighting to balance all groups by age; after weighting, standardized differences among groups ranged from 0.02 to 0.21 (versus 0.12 to 0.85 before weighting). The character and strength of the group separations were strengthened slightly, for PD in particular (the most out-of-balance group by age), but remained essentially unchanged (Supplementary Figure 7). From examination of LD function loadings (Figure 6B), we identified four miRNAs that contribute the most to AD vs. PD vs. DLB classification, with miR-26a-5p and 146a-5p being most important for AD vs. PD + DLB (orange bold) and miR-142-3p and 101-3p being most important for DLB vs. AD+PD (blue bold). Note that miR-101-3p was also identified as a top contributing miRNA to the ENR all 57-miRNA models (Figure 5E). Interestingly, individual target prediction and IPA analysis using the two miRNAs most important for AD discrimination (miR-26a-5p, miR-146a-5p) vs. the two miRNAs most important for DLB discrimination (miR-142-3p, miR-101-3p) identified Neurological Diseases or Functions that were clinically relevant to AD and DLB/PD, respectively, and with minimal overlap (Figure 6C; Table 2; Supplementary Tables 7, 8) as well as clinically relevant Canonical Pathways (Supplementary Tables 9, 10). For instance, within the Neurological Disease category the top 20 functions and diseases predicted to be impacted by the two AD miRNAs but not the two DLB miRNAs include AD, AD or frontotemporal dementia, degenerative dementia, dementia, progressive neurological disorder, and tauopathy (Figure 6C; Table 2A; Supplementary Table 7). In contrast, the predicted targets that were significantly over-represented by the two DLB miRNAs but not the two AD miRNAs include chorea, disorder of the basal ganglia, insomnia, movement disorders, neuromuscular disease, and sleep disorders (Figure 6C; Table 2B; Supplementary Table 8). It should be noted, however, that the association of these miRNAs with sleep disorders is limited to target prediction, as this dataset did not include standardized sleep questionnaires or polysomnography. Cognitive impairment was identified as being regulated by the predicted mRNA targets of both the two AD and two DLB miRNAs. Together, these data demonstrate that subsets of plasma miRNAs discriminate individuals based on the kind of neurodegenerative disease and may also be informative as biomarkers to specific disease symptoms.

**Table 2:** **(A)** Top 20 neurological diseases or functions with significant overrepresentation of the two miRNAs most important for discriminating AD vs. PD and DLB (miR-26a-5p, miR-146a-5p); **(B)** Top 20 neurological diseases or functions with significant overrepresentation of the two miRNAs most important for discriminating DLB vs. AD and PD (miR-142-3p, miR-101-3p).

A. AD: miR-26a-5p and miR-146a-5p		B. DLB: miR-142-3p and miR-101-3p	
Diseases or functions	*p*-value	Diseases or functions	*p*-value
Familial neurological disorder	8.56E-09	Familial neurological disorder	7.89E-06
Familial encephalopathy	9.97E-09	Neuromuscular disease	8.46E-06
Major depression	1.69E-08	Sleep disorders	4.18E-05
Familial CNS disease	2.53E-08	Disorder of basal ganglia	6.09E-05
Schizophrenia	2.09E-07	Familial encephalopathy	1.16E-04
Cognitive impairment	8.50E-07	Insomnia	1.50E-04
Autosomal dominant encephalopathy	8.90E-07	Neurocutaneous syndrome	1.71E-04
Intellectual disability	1.09E-06	Familial CNS disease	1.97E-04
Degenerative dementia	3.58E-06	Syndromic X-linked intellectual disability	2.20E-04
AD or FTD	3.95E-06	X-linked intellectual disability	3.25E-04
Tauopathy	4.25E-06	Hereditary neuromuscular disease	3.32E-04
Progressive encephalopathy	5.74E-06	Chorea	4.77E-04
Progressive neurological disorder	7.00E-06	Movement disorders	5.38E-04
Alzheimer disease	8.86E-06	Cognitive impairment	6.67E-04
Familial intellectual disability	1.09E-05	FG syndrome	7.42E-04
Dementia	1.41E-05	Schizophrenia	8.65E-04
Syndromic intellectual disability	1.43E-05	Congenital encephalopathy	9.01E-04
Pervasive developmental disorder	3.64E-05	Huntington disease	1.00E-03
Seizures	3.66E-05	Congenital neurological disorder	1.36E-03
Seizure disorder	4.21E-05	Dominant intellectual disability	1.53E-03

## 4 Discussion

Current methods to discriminate AD from PD from DLB are challenging due to similarities in their clinical presentations, and more so for those who are afflicted with two of these diseases. For example, the definitive diagnosis of AD from DLB is difficult due to their clinical similarities and the potential for a positive result with plasma AD pathology assays in some DLB patients (Sierra et al., 2016; Alam et al., 2023). Consequently, there is great interest in establishing new plasma biomarkers that add information beyond the hallmark pathologies caused by Aβ, tau, and α-synuclein in order to distinguish AD from PD from DLB. Within the context of diagnosing these diseases from NC, several studies have proposed blood-based panels using combinations of multiple miRNAs (Kumar et al., 2013; Leidinger et al., 2013; Tan et al., 2014; Cheng et al., 2015; Lugli et al., 2015; Ding et al., 2016; Guo et al., 2017; Nagaraj et al., 2017; Denk et al., 2018; Ludwig et al., 2019; Patil et al., 2019; Barbagallo et al., 2020; Cheng et al., 2020; Li et al., 2020; Nie et al., 2020; Oliveira et al., 2020; Zhao et al., 2020; Dong et al., 2021; Jia et al., 2021; Zhou et al., 2021; Abuelezz et al., 2022; Jia et al., 2022; Pena-Bautista et al., 2022; Kumar et al., 2023; Chai et al., 2024; Gutierrez-Tordera et al., 2024; Kruger et al., 2024; Li et al., 2024; Cots et al., 2025; Ravanidis et al., 2025). However, fewer studies have assessed the specificity of blood-based miRNA models for AD vs. PD and/or DLB (Leidinger et al., 2013; Gamez-Valero et al., 2019; Patil et al., 2019; Shigemizu et al., 2019a; Barbagallo et al., 2020; Asanomi et al., 2021; Gamez-Valero et al., 2021; Jia et al., 2021; Li et al., 2022). For example, to our knowledge, six publications to date have examined miRNAs for their differential expression in AD vs. other dementias. Shigemizu et al. (2019a) examined miRNA expression in 1,601 serum samples from Japanese individuals (1,021 AD, 91 VaD, 169 DLB, 32 MCI, 288 NC) to investigate potential miRNA biomarkers and construct risk prediction models, based on a supervised principal component analysis logistic regression method, according to the subtype of dementia. Asanomi et al. (2021), carried out a comprehensive miRNA study in serum from 1,348 Japanese individuals (1,009 AD, 89 VaD, 166 DLB, 84 Normal Pressure Hydrocephalus, 246 NC) to construct dementia subtype prediction models based on penalized regression models with the multiclass classification. Their final prediction model classified dementia patients into four dementia subtypes using 46 miRNAs, and network-based meta-analysis of the miRNA targets revealed several important hub genes associated with the pathogenesis of dementia subtypes (Asanomi et al., 2021). Jia et al. (2021) examined plasma miRNAs from Chinese individuals for a pilot study (23 AD, 21 NC), then a second study (190 AD, 216 NC), then a third study (151 AD, 153 NC) to establish and verify a predictive model of P-tau/Aβ42 in CSF. Their test was then applied to a fourth study (155 AD, 55 amnestic MCI, 51 VaD, 53 PD, 53 behavioral variant FTD, 52 DLB, 139 NC) to assess the diagnostic capacity of miRNAs and they reported that a panel of seven serum miRNAs in can predict P-tau/Aβ42 in CSF and readily differentiate AD from VaD, PD, DLB, and behavioral variant FTD. Li et al. (2022) used a computational analysis approach to examine serum miRNA expression profiles in Japanese individuals with data obtained from the Gene Expression Omnibus database in 1,601 samples (1,021 AD, 91 VaD, 169 DLB, 32 MCI, 288 NC). Their studies identified 2,547 miRNAs in the expression profiles, and they subsequently performed a computational workflow to detect key miRNA features and expression patterns in the expression profiles. They identified an optimal miRNA biomarker set on the basis of the evaluation metrics of classifiers under varying features and reported that the relationship between candidate features including miR-3184-5p, miR-6088, and miR-4649 and neurodegenerative diseases was validated in recent studies, confirming the efficacy of their methods (Li et al., 2022).

While the studies described above demonstrated disease specificity in blood miRNA profiles, only three directly compared the performance of blood-based miRNAs for classification of AD vs. DLB (Gamez-Valero et al., 2019; Asanomi et al., 2021; Gamez-Valero et al., 2021). A multi-miRNA model with seven platelet miRNAs was shown to have robust classification for AD (*n* = 13) from DLB (*n* = 24) (Gamez-Valero et al., 2021). In an exploratory study, two miRNAs in plasma extracellular vesicles robustly classified AD (*n* = 10) from DLB (*n* = 18; AUC = 0.9) (Gamez-Valero et al., 2019). Further, while the Asanomi et al. (2021) study developed a dementia subtype prediction model with 46 serum miRNAs, in this complex sample population the model only achieved a mean accuracy of 0.38 for classifying the five groups. Together this limited but compelling data demonstrates the potential of blood-based miRNAs to aid in the definitive diagnosis of AD from DLB and PD.

Here we used machine learning to develop prediction models for AD vs. PD vs. DLB using human plasma from biorepositories in the United States. For clinical translatability, our study used total plasma collected and processed using standard clinical methods, and RT-qPCR for miRNA analysis. ENR and LD analysis were used to develop predictive models based on 57 miRNAs previously associated with AD from our and other studies (Lusardi et al., 2017; Nagaraj et al., 2017; Qian et al., 2019; Wiedrick et al., 2019; Sandau et al., 2020; Awuson-David et al., 2023; Sandau et al., 2024; Sandau et al., 2025). ENR models achieved good separation of AD from both DLB (cvAUC = 0.77) and PD (cvAUC = 0.80) with a subset of six miRNAs being most important overall (miR-19a-3p, −22-3p, −92b-3p, −101-3p, −143-3p, −423-5p). Of these six, miR-19a-3p and miR-101-3p were identified in a meta-analysis of 30 publications as consistently upregulated in synucleinopathies, and miR-92b-3p as upregulated in amyloidopathies (Noronha et al., 2022). LD analysis also achieved a robust classification of AD from PD + DLB (cvAUC = 0.94), PD from AD+DLB (cvAUC = 0.88), and DLB from AD+PD (cvAUC = 0.85). We also show that miR-26a-5p and -146a-5p were most important for AD vs. PD + DLB and miR-142-3p and −101-3p were most important for DLB vs. AD+PD. MiR-146a-5p is consistently upregulated in both AD and PD, while miR-142-3p is downregulated in amyloidopathies (Noronha et al., 2022). Here, miR-101-3p also ranked high in terms of importance for both the ENR and LD models.

One limitation of this study is the inclusion of samples from multiple sources, with all diagnostic groups confined to particular sites with almost no overlap (only 10 AD samples came from a different site to the other 77 AD samples, all other disease groups being site-specific), such that “site effects” might exaggerate differences between diagnoses. Since the collection and storage methods are identical across sites, these factors are not likely to influence the results, but we acknowledge that differences in storage time or demographics might confound these results. Another critical limitation was the very small DLB sample size (*n* = 20, with only 2 females), which affected our confidence in DLB-related results throughout. It should be noted in the figures that cvAUC SDs when classifying DLB are generally 2 to 3 times larger than the corresponding cvAUC SDs for AD and PD classification. The DLB samples were also found to be highly influential on the sex-specific exploratory analyses we performed, calling the generalizability of those results into serious question.

Some plasma miRNAs prioritized in our prediction models were previously identified as contributors to other models for classifying neurodegenerative diseases. MiR-143-3p and miR-146a-5p are two of seven plasma miRNAs predictive of CSF p-tau and Aβ_42_ measurements in AD, and a model using those predicted p-tau/Aβ_42_ values classified AD vs. pooled NCs plus other neurological diseases (DLB, PDD, VaD, FTD; AUC vascular dementia, frontal temporal dementia; AUC = 0.87; Jia et al., 2021). MiR-146a-3p was proposed as one of three miRNAs that discriminate AD from VaD (Dong et al., 2015). Serum miR-22-3p and miR-26a-5p are two of nine miRNAs informative to mild, moderate, or severe AD (Guo et al., 2017). Serum exosome miR-22-3p was identified as a contributor to a three-miRNA classifier of AD vs. NC (Guo et al., 2017). Our prior studies identified miR-423-5p as a high-priority candidate for AD classification (Sandau et al., 2025). Specifically, we used plasma expression data for the same 57 miRNAs assessed herein and combined information from ENR and Bayesian model-averaging to identify a parsimonious model for AD vs. NC classification (Sandau et al., 2025). MiR-423-5p and miR-23a-3p were top-ranked miRNAs with decreased and increased expression in AD, respectively, and their expression ratio had good performance for AD relative to NC in both the discovery (AUC = 0.771) and validation (AUC = 0.743) cohorts (Sandau et al., 2025). Here we recapitulated the performance of this miRNA classifier for AD vs. NC (cvAUC = 0.75) and show a more robust performance for both PD (cvAUC = 0.86) and DLB (cvAUC = 0.88) vs. NC. Together, our studies and prior publications (1) support the reproducibility of blood-based miRNAs as biomarkers for AD, PD, and/or DLB, and (2) demonstrate the need to consider broader dementia contexts to maintain disease specificity for new diagnostic biomarkers.

We also performed miRNA target prediction and pathway analysis for (1) six miRNAs most important to the ENR analysis, (2) two miRNAs most important for AD vs. PD + DLB classification by LD analysis, and (3) two miRNAs most important for DLB vs. AD+PD classification by LD analysis. For the six ENR miRNAs, the top canonical pathway identified was the Synaptogenesis Signaling pathway with 77 predicted mRNA targets (Supplementary Table 6). This pathway is highly relevant as synaptic dysfunction and synaptic loss is an early feature of neurodegenerative diseases (Subramanian and Tremblay, 2021). Predicted targets within this pathway include multiple glutamate receptors (NMDA receptor subunit 2A, AMPA receptor subunit 1, multiple metabotropic glutamate receptor subtypes), and proteins integral to neurotransmitter vesicle release (synaptobrevin-3, multiple synaptotagmin subtypes) and dendritic spine development (WASP like actin nucleation promoting factor). Other significant canonical pathways of interest returned from the target prediction for those six miRNAs included Amyloid Processing, Autophagy, Neuroinflammation Signaling, and Senescence. Interestingly, a comparison of pathway analyses using the two miRNAs most important for AD discrimination (miR-26a-5p, miR-146a-5p) vs. the two miRNAs most important for DLB discrimination (miR-142-3p, miR-101-3p) identified Neurological Diseases or Functions that are clinically relevant to AD and DLB, respectively (Supplementary Tables 7, 8), with minimal overlap.

These target predictions and pathway analysis results suggest that disease-associated changes in plasma miRNAs reflect neurological changes. In support of this, miR-101-3p (ENR and LD analyses) is implicated in directly regulating amyloid precursor protein via the 3’UTR (Vilardo et al., 2010). MiR-101-3p is upregulated in the substantia nigra of postmortem human PD brain tissue and contributes to α-synuclein aggregation and toxicity in cultured neurons (Zhang et al., 2021). MiR-19a-3p (ENR analysis) was previously shown to be decreased in the dorsolateral prefrontal cortex of donors with DLB pathology, while miR-423-5p (ENR analysis) was associated with atherosclerosis (Luo et al., 2025). In an independent analysis of our reposited plasma miRNA data (Sandau et al., 2025), Han et al. (2025) used principal component analysis to demonstrate that decreases in plasma miR-423-5p in AD participants were associated with increases in amyloid PET, and decreases in hippocampal volume, baseline memory and executive function, and longitudinal changes in memory and executive function. Interestingly, participant classification using our LD models, which assigned a lower priority to miR-423-5p, was not associated with MMSE scores, suggesting that the miRNAs most important to the LD models may be informative for diagnosing neurodegenerative disease subtypes regardless of cognitive state, while models that are more dependent on miR-423-5p may aid in monitoring disease progression. However, future studies in large patient populations with a broad range of scores across cognitive tests are needed to more definitively assess the relationship to plasma miRNA levels.

In conclusion, our findings and prior studies support that plasma miRNAs may aid in the differential diagnosis and/or serve as biomarkers for clinical features of AD, PD, and DLB. Future studies that assess plasma miRNA models in combination with p-tau217/Aβ_42_ plasma ratio measurements (Hu et al., 2025) may identify an optimal blood-based assay with boosted performance compared to either standalone measure. Both here and in our prior studies, we also show results that hint at sex-dependent effects on plasma miRNAs (Sandau et al., 2024; Sandau et al., 2025). However, it was not possible to reliably estimate sex-dependent effects in miRNA expression within AD, PD, and/or DLB due to sample size limitations. Another limitation to our study is that we did not analyze associations between the performance of plasma miRNA models and relevant co-morbidities, such as cardiovascular disease. Further, here we relied on scores to assess the severity of cognitive impairment in PD, and to a lesser extent, DLB. However, the PD group had a wide range of cognitive function. A recent study showed that while significant α-synuclein pathology is the main substrate of dementia in PD, coexistent pathologies are common. In particular, Aβ and tau pathologies independently contribute to the development and pattern of cognitive decline in PD (Smith et al., 2019). In this regard, the PANUC PD patients are unique in that they underwent a comprehensive cognitive test battery, then received an additional cognitive diagnosis: PD-NCI, PD-MCI, and PDD. While incorporating cognitive scores into patient analyses is challenging as each cohort underwent a different battery of tests, here we assessed the performance of the LD models in these subgroups. Discrimination was most similar to the combined PD group for the PD-NCI (long-dash line, closest to the “PD” solid line) while the PD-MCI (medium-dash) and PDD (short-dash) classified less similar to the combined PD and had more overlap with DLB (Supplementary Figure 8). Of note, one participant diagnosed with PD at the time of sample procurement in 2011 was diagnosed with progressive supranuclear palsy in 2025 based on clinical symptoms (Supplementary Figure 8, black diamond). These findings underscore that plasma miRNAs can add information to clinical diagnostics and demonstrate the need for larger-scale studies to validate prediction models across more diverse and representative patient populations.

## Data Availability

All data used for statistical comparisons in this study, the amplification flag summary by miRNA and experimental group used to select miRNAs for differential expression or differential detection, and the datasets generated for this study are publicly available in the Gene Expression Omnibus Accession site: https://www.ncbi.nlm.nih.gov/geo/query/acc.cgi?acc=GSE338073.

## Footnotes

1
adni.loni.usc.edu

2
https://assets.thermofisher.com/TFS-Assets/GSD/Reference-Materials/identifying-mirna-normalizers-white-paper.pdf

## Supplementary material

SUPPLEMENTARY FIGURE 1 Correcting miRNA qPCR assay variance with sequential calibrations and normalization. Median level of (1) raw Cq values of all 64 miRNAs included in the analysis (gray line), (2) the Cq values following technical calibration with cel-miR-39-3p (light blue line), (3) the technical-calibrated Cq values following sequential calibration by batch and run sequence (light blue line), and (4) the calibrated (technical and batch) Cq values following normalization to endogenous control miRNAs (blue line).

SUPPLEMENTARY FIGURE 2 Separation of NC, AD, PD, and DLB using nonmetric multidimensional scaling with all 57 miRNAs as information. Multidimensional scaling (MDS) projection of the miRNA expression space into two dimensions using distances derived from a random forest classifier of the diagnosis groups (NC=green, AD=orange, PD=pink, DLB=blue). Lines of best separation for each group vs. all the others were calculated via logistic regressions on the MDS coordinates. Mean area under the receiver operating characteristic curve (cvAUC) and standard deviation (SD) of cvAUC were calculated by 7-fold cross-validation, using the same 7-fold divisions for all group comparisons. Interestingly, NC was the most separable group with respect to the two axes, and AD and PD cluster as “steps” along the same vector of larger distance from NC; that vector is not orthogonal to either coordinate axis, hinting at higher-order complexity in the information space. DLB is nearly overlaid on PD in this space, tending to show up on the far extremity of the “PD” region but without a distinct region of its own. See also Figure 6A.

SUPPLEMENTARY FIGURE 3 Distribution of cognitive status for NC, AD, PD and DLB participants. Relative density plot showing the distributions of the Mini-Mental State Examination (MMSE) scores for NC, AD, PD, and DLB groups. Note that for the PD and DLB groups the plotted values are an equivalent weighted MMSE score that was converted from raw Montreal Cognitive Assessment (MoCA) scores. The NC and AD values are based on MMSE assessment.

SUPPLEMENTARY FIGURE 4 Categorization of miRNAs nonspecific to disease state. **(A)** Group contrasts of the 24 miRNAs nonspecific for disease state based on the AD-specificity score for NC vs AD, NC vs PD, NC vs DLB, and NC vs All {AD+PD+DLB}. The cell values, hue (negative=blue, positive=red), and color intensity reflect the z-score of the corresponding contrast from the miRNA expression association models. The cell size is proportional to the standardized absolute contrast magnitude. **(B,C)** Relative density plots showing the distribution of participants within the NC (green), AD (yellow), PD (pink), and DLB (blue) groups for the expression levels of a representative miRNA that was **(B)** differentially expressed in AD, PD and DLB vs NC (miR-423-5p) and **(C)** not differentially expressed in any group (miR-660-5p). Vertical dashed lines represent the cutpoint for miRNA expression levels where the higher density of AD and NC participants were on opposite sides.

SUPPLEMENTARY FIGURE 5 Sex-dependent differences in plasma miRNA expression among AD, PD, and DLB participants. **(A)** Representative miRNA (miR-19a-3p) where diagnosis group differences were ~3x larger for females than for males. **(B)** Rare representative miRNA (miR-885-5p) where diagnosis group differences were ~2x larger for males than for females.

SUPPLEMENTARY FIGURE 6 Performance of a 4-miRNA “big-effects only” model for classification of AD, PD, and DLB. **(A)** Receiver operating characteristic (ROC) curves for each of the disease groups (AD, PD, DLB) vs NC using the corresponding model-derived predicted probability as the group classifier. Mean area under the ROC curve (cvAUC) and standard deviation (SD) of cvAUC were calculated by 7-fold cross-validation, using the same 7-fold divisions for all group comparisons. All groups show similar separation (cvAUC ~0.8) from NC. **(B)** Relative density plots showing the distributions of the model-derived predicted probabilities for each of the diagnosis groups based on a ridge logistic regression for AD vs NC (only), using the 4 miRNAs (miR-143-3p, miR-145-5p, miR-34a-5p, and miR-584-5p) that demonstrated the largest absolute magnitudes of effect for AD vs NC in individual-miRNA models, adjusted for age and sex. **(C)** ROC curves for AD vs PD, DLB, and {PD+DLB} (pooled, olive-gray) using the corresponding model-derived predicted probability as the group classifier, with cvAUC ± SD calculated by 7-fold cross-validation as above. The “big effects only” classifier achieves poor separation of disease groups from one another.

SUPPLEMENTARY FIGURE 7 Sensitivity analysis of residual age differences between AD, PD, and DLB participants in the LD analysis using 57 miRNAs. LD analysis using 57 miRNAs. LD analysis using propensity-score weighting to balance all groups by age and leave-one-out cross-validated linear discrimination of expression profiles on 57 miRNAs. Mean area under the receiver operating characteristic curve (cvAUC) and standard deviation (SD) of cvAUC were calculated. After weighting, standardized differences among groups ranged from 0.02 to 0.21 (versus 0.12 to 0.85 before weighting, see Figure 6).

SUPPLEMENTARY FIGURE 8 Separation of AD, DLB, PD-NCI, PD-MCI, and PDD participants using the 57 miRNA model. Classification of each study participant by LD analysis using using proportional prior probabilities for diagnosis group membership (AD, orange circle; DLB, blue circle; PD-NCI, pink diamond; PD-MCI; pink arrow; PDD, pink triangle) and leave-one-out cross-validation of the functional parameter estimation was used to generate the two-dimensional functional space through which straight lines were drawn to optimally separate the groups within the space. Mean area under the receiver operating characteristic curve (cvAUC) and standard deviation (SD) of cvAUC were calculated by 7-fold cross-validation, using the same 7-fold divisions for all group comparisons. Note one participant was diagnosed as PD upon sample collection in 2011 then the diagnosis was updated to progressive supranuclear palsy in 2025 based on clinical symptoms (black diamond).

SUPPLEMENTARY TABLE 1A MiRNA array characteristics.

SUPPLEMENTARY TABLE 1B miRBase table.

SUPPLEMENTARY TABLE 1C NTC vs mean CT.

SUPPLEMENTARY TABLE 2 miRNA ICC.

SUPPLEMENTARY TABLE 3 AD-specificity scores.

SUPPLEMENTARY TABLE 4 Sex-dependent effects.

SUPPLEMENTARY TABLE 5 Neuro function-6 miRNA.

SUPPLEMENTARY TABLE 6 Canonical path-6 miRNA.

SUPPLEMENTARY TABLE 7 Neuro function-2 AD miRNA.

SUPPLEMENTARY TABLE 8 Neuro function-2 DLB miRNA.

SUPPLEMENTARY TABLE 9 Canonical Path-2 AD miRNA.

SUPPLEMENTARY TABLE 10 Canonical Path-2 DLB miRNA.

## References

[ref1] Abuelezz N. Z. Nasr F. E. Abdel Aal W. M. Molokhia T. Zaky A. (2022). Sera miR-34a, miR-29b and miR-181c as potential novel diagnostic biomarker panel for Alzheimers in the Egyptian population. Exp. Gerontol. 169:111961. doi: 10.1016/j.exger.2022.111961, 36155067

[ref2] Aisen P. S. Donohue M. C. Raman R. Rafii M. S. Petersen R. C. (2024). The Alzheimer's disease neuroimaging initiative clinical core. Alzheimers Dement. 20, 7361–7368. doi: 10.1002/alz.14167, 39136045 PMC11485391

[ref3] Alam J. J. Maruff P. Doctrow S. R. Chu H. M. Conway J. Gomperts S. N. . (2023). Association of plasma phosphorylated tau with the response to neflamapimod treatment in patients with dementia with Lewy bodies. Neurology 101, e1708–e1717. doi: 10.1212/WNL.0000000000207755, 37657939 PMC10624490

[ref4] Arnold M. R. Coughlin D. G. Brumbach B. H. Smirnov D. S. Concha-Marambio L. Farris C. M. . (2022). Alpha-synuclein seed amplification in CSF and brain from patients with different brain distributions of pathological alpha-synuclein in the context of co-pathology and non-LBD diagnoses. Ann. Neurol. 92, 650–662. doi: 10.1002/ana.26453, 35808984 PMC9489647

[ref5] Asanomi Y. Shigemizu D. Akiyama S. Sakurai T. Ozaki K. Ochiya T. . (2021). Dementia subtype prediction models constructed by penalized regression methods for multiclass classification using serum microRNA expression data. Sci. Rep. 11:20947. doi: 10.1038/s41598-021-00424-1, 34686734 PMC8536697

[ref6] Awuson-David B. Williams A. C. Wright B. Hill L. J. Di Pietro V. (2023). Common microRNA regulated pathways in Alzheimer's and Parkinson's disease. Front. Neurosci. 17:1228927. doi: 10.3389/fnins.2023.1228927, 37719162 PMC10502311

[ref7] Barbagallo C. Mostile G. Baglieri G. Giunta F. Luca A. Raciti L. . (2020). Specific signatures of serum miRNAs as potential biomarkers to discriminate clinically similar neurodegenerative and vascular-related diseases. Cell. Mol. Neurobiol. 40, 531–546. doi: 10.1007/s10571-019-00751-y, 31691877 PMC11448951

[ref8] Bekris L. M. Lutz F. Montine T. J. Yu C. E. Tsuang D. Peskind E. R. . (2013). MicroRNA in Alzheimer's disease: an exploratory study in brain, cerebrospinal fluid and plasma. Biomarkers 18, 455–466. doi: 10.3109/1354750X.2013.814073, 23822153 PMC3967870

[ref9] Blount G. S. Coursey L. Kocerha J. (2022). MicroRNA networks in cognition and dementia. Cells 11. doi: 10.3390/cells11121882, 35741010 PMC9221254

[ref10] Burgos K. Malenica I. Metpally R. Courtright A. Rakela B. Beach T. . (2014). Profiles of extracellular miRNA in cerebrospinal fluid and serum from patients with Alzheimer's and Parkinson's diseases correlate with disease status and features of pathology. PLoS One 9:e94839. doi: 10.1371/journal.pone.0094839, 24797360 PMC4010405

[ref11] Chai Y. L. Strohm L. Zhu Y. Chia R. S. L. Chong J. R. Suresh D. D. . (2024). Extracellular vesicle-enriched miRNA-biomarkers show improved utility for detecting Alzheimer's disease dementia and medial temporal atrophy. J. Alzheimer's Dis 99, 1317–1331. doi: 10.3233/JAD-230572, 38788066 PMC11191453

[ref12] Cheng L. Doecke J. D. Sharples R. A. Villemagne V. L. Fowler C. J. Rembach A. . (2015). Prognostic serum miRNA biomarkers associated with Alzheimer's disease shows concordance with neuropsychological and neuroimaging assessment. Mol. Psychiatry 20, 1188–1196. doi: 10.1038/mp.2014.127, 25349172

[ref13] Cheng L. Vella L. J. Barnham K. J. McLean C. Masters C. L. Hill A. F. (2020). Small RNA fingerprinting of Alzheimer's disease frontal cortex extracellular vesicles and their comparison with peripheral extracellular vesicles. J. Extracell. Vesicles 9:1766822. doi: 10.1080/20013078.2020.1766822, 32922692 PMC7448944

[ref14] Christenson P. R. Jeong H. Li M. Ahn H. Schmeichel A. M. Misra P. . (2024). Blood-based nanoparticle-enhanced quaking-induced conversion (Nano-QuIC): inhibitor-resistant detection of seeding activity in patients diagnosed with Parkinson's disease. Nano Lett. 24, 15016–15024. doi: 10.1021/acs.nanolett.4c03768, 39485864

[ref15] Cohen J. (1988). Statistical Power Analysis for the Behavioral Sciences. New York: Routledge.

[ref16] Cots A. Riberas-Sanchez A. Pericot I. Turon A. Garcia-Brito S. Garre-Olmo J. . (2025). A plasma miRNA signature, including miR-495, as early diagnostic biomarkers associated with cognitive decline in Alzheimer's disease. Alzheimers Dement (Amst) 17:e70213. doi: 10.1002/dad2.70213, 41189611 PMC12580240

[ref17] Denk J. Oberhauser F. Kornhuber J. Wiltfang J. Fassbender K. Schroeter M. L. . (2018). Specific serum and CSF microRNA profiles distinguish sporadic behavioural variant of frontotemporal dementia compared with Alzheimer patients and cognitively healthy controls. PLoS One 13:e0197329. doi: 10.1371/journal.pone.0197329, 29746584 PMC5945001

[ref18] Ding H. Huang Z. Chen M. Wang C. Chen X. Chen J. . (2016). Identification of a panel of five serum miRNAs as a biomarker for Parkinson's disease. Parkinsonism Relat. Disord. 22, 68–73. doi: 10.1016/j.parkreldis.2015.11.014, 26631952

[ref19] Dong Z. Gu H. Guo Q. Liang S. Xue J. Yao F. . (2021). Profiling of serum exosome MiRNA reveals the potential of a MiRNA panel as diagnostic biomarker for Alzheimer's disease. Mol. Neurobiol. 58, 3084–3094. doi: 10.1007/s12035-021-02323-y, 33629272

[ref20] Dong H. Li J. Huang L. Chen X. Li D. Wang T. . (2015). Serum MicroRNA profiles serve as novel biomarkers for the diagnosis of Alzheimer's disease. Dis. Markers 2015:625659. doi: 10.1155/2015/625659, 26078483 PMC4452867

[ref21] Fasnacht J. S. Wueest A. S. Berres M. Thomann A. E. Krumm S. Gutbrod K. . (2023). Conversion between the Montreal cognitive assessment and the Mini-mental status examination. J. Am. Geriatr. Soc. 71, 869–879. doi: 10.1111/jgs.18124, 36346002

[ref22] Gamez-Valero A. Campdelacreu J. Vilas D. Ispierto L. Gascon-Bayarri J. Rene R. . (2021). Platelet miRNA biosignature discriminates between dementia with Lewy bodies and Alzheimer's disease. Biomedicine 9. doi: 10.3390/biomedicines9091272, 34572457 PMC8466211

[ref23] Gamez-Valero A. Campdelacreu J. Vilas D. Ispierto L. Rene R. Alvarez R. . (2019). Exploratory study on microRNA profiles from plasma-derived extracellular vesicles in Alzheimer's disease and dementia with Lewy bodies. Transl Neurodegener 8:31. doi: 10.1186/s40035-019-0169-5, 31592314 PMC6775659

[ref24] Guo R. Fan G. Zhang J. Wu C. Du Y. Ye H. . (2017). A 9-microRNA signature in serum serves as a noninvasive biomarker in early diagnosis of Alzheimer's disease. J. Alzheimer's Dis 60, 1365–1377. doi: 10.3233/JAD-170343, 29036818

[ref25] Gutierrez-Tordera L. Papandreou C. Novau-Ferre N. Garcia-Gonzalez P. Rojas M. Marquie M. . (2024). Exploring small non-coding RNAs as blood-based biomarkers to predict Alzheimer's disease. Cell Biosci. 14:8. doi: 10.1186/s13578-023-01190-5, 38229129 PMC10790437

[ref26] Han S. W. Park Y. H. Pyun J. M. Bice P. J. Kim S. Saykin A. J. . (2025). miR-423-5p and miR-92a-3p in Alzheimer's disease: relationship with pathology and cognition. Front. Aging Neurosci. 17:1637368. doi: 10.3389/fnagi.2025.1637368, 40766177 PMC12321855

[ref27] Hebert S. S. Wang W. X. Zhu Q. Nelson P. T. (2013). A study of small RNAs from cerebral neocortex of pathology-verified Alzheimer's disease, dementia with lewy bodies, hippocampal sclerosis, frontotemporal lobar dementia, and non-demented human controls. J. Alzheimer's Dis 35, 335–348. doi: 10.3233/JAD-122350, 23403535 PMC3753694

[ref28] Hu S. Yu H. Gao J. (2025). The pTau217/Abeta(1-42) plasma ratio: the first FDA-cleared blood biomarker test for diagnosis of Alzheimer's disease. Drug Discov. Ther. 19, 208–209. doi: 10.5582/ddt.2025.01055, 40582836

[ref29] Jack C. R. Jr. Albert M. S. Knopman D. S. McKhann G. M. Sperling R. A. Carrillo M. C. . (2011). Introduction to the recommendations from the National Institute on Aging-Alzheimer's Association workgroups on diagnostic guidelines for Alzheimer's disease. Alzheimers Dement. 7, 257–262. doi: 10.1016/j.jalz.2011.03.004, 21514247 PMC3096735

[ref30] Jia L. Zhu M. Yang J. Pang Y. Wang Q. Li Y. . (2021). Prediction of P-tau/Abeta42 in the cerebrospinal fluid with blood microRNAs in Alzheimer's disease. BMC Med. 19:264. doi: 10.1186/s12916-021-02142-x, 34775974 PMC8591889

[ref31] Jia L. Zhu M. Yang J. Pang Y. Wang Q. Li T. . (2022). Exosomal microRNA-based predictive model for preclinical Alzheimer's disease: a multicenter study. Biol. Psychiatry 92, 44–53. doi: 10.1016/j.biopsych.2021.12.015, 35221095

[ref32] Kapplingattu S. V. Bhattacharya S. Adlakha Y. K. (2025). MiRNAs as major players in brain health and disease: current knowledge and future perspectives. Cell Death Discov. 11:7. doi: 10.1038/s41420-024-02283-x, 39805813 PMC11729916

[ref33] Kruger D. M. Pena-Centeno T. Liu S. Park T. Kaurani L. Pradhan R. . (2024). The plasma miRNAome in ADNI: signatures to aid the detection of at-risk individuals. Alzheimers Dement. 20, 7479–7494. doi: 10.1002/alz.14157, 39291752 PMC11567822

[ref34] Kumar P. Dezso Z. MacKenzie C. Oestreicher J. Agoulnik S. Byrne M. . (2013). Circulating miRNA biomarkers for Alzheimer's disease. PLoS One 8:e69807. doi: 10.1371/journal.pone.0069807, 23922807 PMC3726785

[ref35] Kumar A. Su Y. Sharma M. Singh S. Kim S. Peavey J. J. . (2023). MicroRNA expression in extracellular vesicles as a novel blood-based biomarker for Alzheimer's disease. Alzheimers Dement. 19, 4952–4966. doi: 10.1002/alz.13055, 37071449 PMC11663460

[ref36] Leidinger P. Backes C. Deutscher S. Schmitt K. Mueller S. C. Frese K. . (2013). A blood based 12-miRNA signature of Alzheimer disease patients. Genome Biol. 14:R78. doi: 10.1186/gb-2013-14-7-r78, 23895045 PMC4053778

[ref37] Li Y. Cao Y. Liu W. Chen F. Zhang H. Zhou H. . (2024). Candidate biomarkers of EV-microRNA in detecting REM sleep behavior disorder and Parkinson's disease. NPJ Parkinsons Dis 10:18. doi: 10.1038/s41531-023-00628-4, 38200052 PMC10781790

[ref38] Li Z. Guo W. Ding S. Chen L. Feng K. Huang T. . (2022). Identifying key MicroRNA signatures for neurodegenerative diseases with machine learning methods. Front. Genet. 13:880997. doi: 10.3389/fgene.2022.880997, 35528544 PMC9068882

[ref39] Li F. Xie X. Y. Sui X. F. Wang P. Chen Z. Zhang J. B. (2020). Profile of pathogenic proteins and MicroRNAs in plasma-derived extracellular vesicles in Alzheimer's disease: a pilot study. Neuroscience 432, 240–246. doi: 10.1016/j.neuroscience.2020.02.044, 32135232

[ref40] Ludwig N. Fehlmann T. Kern F. Gogol M. Maetzler W. Deutscher S. . (2019). Machine learning to detect Alzheimer's disease from circulating non-coding RNAs. Genomics Proteomics Bioinformatics 17, 430–440. doi: 10.1016/j.gpb.2019.09.004, 31809862 PMC6943763

[ref41] Lugli G. Cohen A. M. Bennett D. A. Shah R. C. Fields C. J. Hernandez A. G. . (2015). Plasma Exosomal miRNAs in persons with and without Alzheimer disease: altered expression and prospects for biomarkers. PLoS One 10:e0139233. doi: 10.1371/journal.pone.0139233, 26426747 PMC4591334

[ref42] Luo T. Vattathil S. M. Lori A. Schneider J. A. Bennett D. A. Wingo T. S. . (2025). Brain microRNAs differentially expressed in age-related cerebral pathologies. Neurobiol. Aging 151, 42–53. doi: 10.1016/j.neurobiolaging.2025.03.014, 40228357 PMC13333544

[ref43] Lusardi T. A. Phillips J. I. Wiedrick J. T. Harrington C. A. Lind B. Lapidus J. A. . (2017). MicroRNAs in human cerebrospinal fluid as biomarkers for Alzheimer's disease. J. Alzheimer's Dis 55, 1223–1233. doi: 10.3233/JAD-160835, 27814298 PMC5587208

[ref44] McKeith I. G. Boeve B. F. Dickson D. W. Halliday G. Taylor J. P. Weintraub D. . (2017). Diagnosis and management of dementia with Lewy bodies: fourth consensus report of the DLB consortium. Neurology 89, 88–100. doi: 10.1212/WNL.0000000000004058, 28592453 PMC5496518

[ref45] Nagaraj S. Laskowska-Kaszub K. Debski K. J. Wojsiat J. Dabrowski M. Gabryelewicz T. . (2017). Profile of 6 microRNA in blood plasma distinguish early stage Alzheimer's disease patients from non-demented subjects. Oncotarget 8, 16122–16143. doi: 10.18632/oncotarget.15109, 28179587 PMC5369952

[ref46] Nelson P. T. Wang W. X. Janse S. A. Thompson K. L. (2018). MicroRNA expression patterns in human anterior cingulate and motor cortex: a study of dementia with Lewy bodies cases and controls. Brain Res. 1678, 374–383. doi: 10.1016/j.brainres.2017.11.009, 29146111 PMC5752138

[ref47] Nie C. Sun Y. Zhen H. Guo M. Ye J. Liu Z. . (2020). Differential expression of plasma exo-miRNA in neurodegenerative diseases by next-generation sequencing. Front. Neurosci. 14:438. doi: 10.3389/fnins.2020.00438, 32457573 PMC7227778

[ref48] Nihashi T. Ito K. Terasawa T. (2020). Diagnostic accuracy of DAT-SPECT and MIBG scintigraphy for dementia with Lewy bodies: an updated systematic review and Bayesian latent class model meta-analysis. Eur. J. Nucl. Med. Mol. Imaging 47, 1984–1997. doi: 10.1007/s00259-019-04480-8, 31423561

[ref49] Noronha O. Mesarosovo L. Anink J. J. Iyer A. Aronica E. Mills J. D. (2022). Differentially expressed miRNAs in age-related neurodegenerative diseases: a meta-analysis. Genes (Basel) 13. doi: 10.3390/genes13061034, 35741796 PMC9222420

[ref50] Oliveira S. R. Dionisio P. A. Correia Guedes L. Goncalves N. Coelho M. Rosa M. M. . (2020). Circulating inflammatory miRNAs associated with Parkinson's disease pathophysiology. Biomolecules 10. doi: 10.3390/biom10060945, 32585840 PMC7356527

[ref51] Parast L. Hunt P. Griffin B. A. Powell D. (2020). When is a match sufficient? A score-based balance metric for the synthetic control method. J. Causal Infer. 8, 209–228. doi: 10.1515/jci-2020-0013

[ref52] Patil K. S. Basak I. Dalen I. Hoedt E. Lange J. Lunde K. A. . (2019). Combinatory microRNA serum signatures as classifiers of Parkinson's disease. Parkinsonism Relat. Disord. 64, 202–210. doi: 10.1016/j.parkreldis.2019.04.010, 31003905

[ref53] Pena-Bautista C. Tarazona-Sanchez A. Braza-Boils A. Balaguer A. Ferre-Gonzalez L. Canada-Martinez A. J. . (2022). Plasma microRNAs as potential biomarkers in early Alzheimer disease expression. Sci. Rep. 12:15589. doi: 10.1038/s41598-022-19862-6, 36114255 PMC9481579

[ref54] Postuma R. B. Poewe W. Litvan I. Lewis S. Lang A. E. Halliday G. . (2018). Validation of the MDS clinical diagnostic criteria for Parkinson's disease. Mov. Disord. 33, 1601–1608. doi: 10.1002/mds.27362, 30145797

[ref55] Qian Q. Zhang J. He F. P. Bao W. X. Zheng T. T. Zhou D. M. . (2019). Down-regulated expression of microRNA-338-5p contributes to neuropathology in Alzheimer's disease. FASEB J. 33, 4404–4417. doi: 10.1096/fj.201801846R, 30576233 PMC6404576

[ref56] Quinn J. F. Gray N. E. (2024). Fluid biomarkers in dementia diagnosis. Continuum (Minneap Minn) 30, 1790–1800. doi: 10.1212/CON.0000000000001497 39620844

[ref57] Ravanidis S. Bougea A. Koros C. Simitsi A. M. Kokotis P. Stefanis L. . (2025). Plasma miRNA biomarker signatures in parkinsonian syndromes. Mol. Neurobiol. 62, 10118–10132. doi: 10.1007/s12035-025-04890-w, 40184025 PMC12289787

[ref58] Sandau U. S. Wiedrick J. T. McFarland T. J. Chen S. Quinn J. F. Saugstad J. A. (2025). Comparison of multi-miRNA models from donor-matched plasma and cerebrospinal fluid as candidate biomarkers for Alzheimer's disease prediction. Sci. Rep. 16:3207. doi: 10.1038/s41598-025-33589-0, 41444408 PMC12830807

[ref59] Sandau U. S. Wiedrick J. T. McFarland T. J. Galasko D. R. Fanning Z. Quinn J. F. . (2024). Analysis of the longitudinal stability of human plasma miRNAs and implications for disease biomarkers. Sci. Rep. 14:2148. doi: 10.1038/s41598-024-52681-5, 38272952 PMC10810819

[ref60] Sandau U. S. Wiedrick J. T. Smith S. J. McFarland T. J. Lusardi T. A. Lind B. . (2020). Performance of validated MicroRNA biomarkers for Alzheimer's disease in mild cognitive impairment. J. Alzheimer's Dis 78, 245–263. doi: 10.3233/JAD-200396, 32955460 PMC9262405

[ref61] Shigemizu D. Akiyama S. Asanomi Y. Boroevich K. A. Sharma A. Tsunoda T. . (2019a). Risk prediction models for dementia constructed by supervised principal component analysis using miRNA expression data. Commun Biol 2:77. doi: 10.1038/s42003-019-0324-7, 30820472 PMC6389908

[ref62] Shigemizu D. Akiyama S. Asanomi Y. Boroevich K. A. Sharma A. Tsunoda T. . (2019b). A comparison of machine learning classifiers for dementia with Lewy bodies using miRNA expression data. BMC Med. Genet. 12:150. doi: 10.1186/s12920-019-0607-3, 31666070 PMC6822471

[ref63] Sierra M. Gelpi E. Marti M. J. Compta Y. (2016). Lewy- and Alzheimer-type pathologies in midbrain and cerebellum across the Lewy body disorders spectrum. Neuropathol. Appl. Neurobiol. 42, 451–462. doi: 10.1111/nan.12308, 26810462

[ref64] Smith C. Malek N. Grosset K. Cullen B. Gentleman S. Grosset D. G. (2019). Neuropathology of dementia in patients with Parkinson's disease: a systematic review of autopsy studies. J. Neurol. Neurosurg. Psychiatry 90, 1234–1243. doi: 10.1136/jnnp-2019-321111, 31444276

[ref65] Sorensen S. S. Nygaard A. B. Christensen T. (2016). miRNA expression profiles in cerebrospinal fluid and blood of patients with Alzheimer's disease and other types of dementia - an exploratory study. Transl Neurodegener 5:6. doi: 10.1186/s40035-016-0053-5, 26981236 PMC4791887

[ref66] Subramanian J. Tremblay M. E. (2021). Editorial: synaptic loss and neurodegeneration. Front. Cell. Neurosci. 15:681029. doi: 10.3389/fncel.2021.681029, 33967701 PMC8097031

[ref67] Tan L. Yu J. T. Tan M. S. Liu Q. Y. Wang H. F. Zhang W. . (2014). Genome-wide serum microRNA expression profiling identifies serum biomarkers for Alzheimer's disease. J. Alzheimer's Dis 40, 1017–1027. doi: 10.3233/JAD-132144, 24577456

[ref68] Vilardo E. Barbato C. Ciotti M. Cogoni C. Ruberti F. (2010). MicroRNA-101 regulates amyloid precursor protein expression in hippocampal neurons. J. Biol. Chem. 285, 18344–18351. doi: 10.1074/jbc.M110.112664, 20395292 PMC2881760

[ref69] Wang W. X. Huang Q. Hu Y. Stromberg A. J. Nelson P. T. (2011). Patterns of microRNA expression in normal and early Alzheimer's disease human temporal cortex: white matter versus gray matter. Acta Neuropathol. 121, 193–205. doi: 10.1007/s00401-010-0756-0, 20936480 PMC3073518

[ref70] Wiedrick J. T. Phillips J. I. Lusardi T. A. McFarland T. J. Lind B. Sandau U. S. . (2019). Validation of MicroRNA biomarkers for Alzheimer's disease in human cerebrospinal fluid. J. Alzheimer's Dis 67, 875–891. doi: 10.3233/JAD-180539, 30689565 PMC6687305

[ref71] Willis A. W. Roberts E. Beck J. C. Fiske B. Ross W. Savica R. . (2022). Incidence of Parkinson disease in North America. NPJ Parkinsons Dis 8:170. doi: 10.1038/s41531-022-00410-y, 36522332 PMC9755252

[ref72] Zhang M. Liu W. Zhang Q. Hu H. (2021). miR-101-3p contributes to alpha-Synuclein aggregation in neural cells through the miR-101-3p/SKP1/PLK2 pathway. J Healthc Eng 2021:6147434. doi: 10.1155/2021/6147434, 34234930 PMC8216813

[ref73] Zhao X. Kang J. Svetnik V. Warden D. Wilcock G. David Smith A. . (2020). A machine learning approach to identify a circulating MicroRNA signature for Alzheimer disease. J Appl Lab Med 5, 15–28. doi: 10.1373/jalm.2019.029595, 31811079

[ref74] Zhou S. Meng Q. Li L. Hai L. Wang Z. Li Z. . (2021). Identification of a qualitative signature for the diagnosis of dementia with Lewy bodies. Front. Genet. 12:758103. doi: 10.3389/fgene.2021.758103, 34868234 PMC8640079

