# Supplementary material for: Plasma multi-miRNA models classify Alzheimer’s, Parkinson’s, and Lewy body dementia
Source: Front Aging Neurosci. 2026 Aug 11;18:1788991. doi: 10.3389/fnagi.2026.1788991 (PMC13503333; doi:10.3389/fnagi.2026.1788991)
Supplement: Supplementary file 1 [file Data_Sheet_1.pdf]

Resource identification initiative	
**Antibody or tool/resource**	**RRID**
Bio-Rad T100 PCR	SCR_021921
ExpressionSuite Software	SCR_021095
Stata version 17.0 software	SCR_012763
Ingenuity Pathway Analysis	SCR_008653
miRBase	SCR_003152
miRDB	SCR_010848
Orbitor RS2 Microplate Robot	SCR_020721
QuantStudio™ 12 K Flex Real-Time PCR System	SCR_021098
QuantStudio™ 12 K Flex Real-Time PCR Software	SCR_021096
TargetScan v.7.2	SCR_010845
